# Neutrophil-Enriched Biomarkers and Long-Term Prognosis in Acute Coronary Syndrome: a Systematic Review and Meta-analysis

**DOI:** 10.1007/s12265-023-10425-2

**Published:** 2023-08-18

**Authors:** Jaquelina Y. T. Yiu, Kathryn E. Hally, Peter D. Larsen, Ana S. Holley

**Affiliations:** Wellington Cardiovascular Research Group, Department of Surgery & Anaesthesia, University of Otago, PO Box 7343, Wellington, New Zealand

**Keywords:** Neutrophils, Acute coronary syndromes, Prognosis

## Abstract

Activated neutrophils release a range of inflammatory products that represent potential biomarkers, and there is interest in the prognostic value of these in acute coronary syndrome (ACS) patients. We conducted a systematic review to examine neutrophil-enriched biomarkers and the occurrence of major adverse cardiovascular events (MACE) in patients with ACS. We identified twenty-seven studies including 17,831 patients with ACS. The most studied biomarkers were neutrophil gelatinase-associated lipocalin (NGAL) and myeloperoxidase (MPO). Meta-analyses showed that elevated NGAL was associated with higher MACE rates (unadjusted risk ratio (RR) 1.52, 95% CI 1.12–2.06, *p* = 0.006) as were elevated MPO levels (unadjusted RR 1.61, 95% CI 1.22–2.13, *p* = 0.01). There was limited data suggesting that increased levels of calprotectin, proteinase-3 and double-stranded DNA were also associated with MACE. These results suggest that higher levels of neutrophil-enriched biomarkers may be predictive of MACE in patients with ACS, although higher-quality studies are needed to confirm these observations.

**Graphical abstract:**

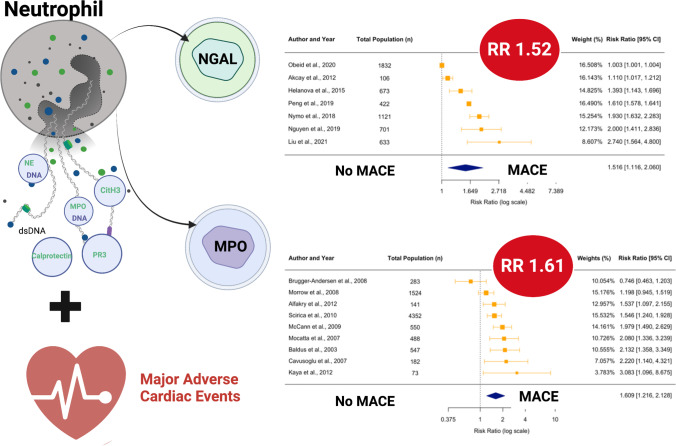

**Supplementary Information:**

The online version contains supplementary material available at 10.1007/s12265-023-10425-2.

## Introduction

Acute coronary syndrome (ACS) is one of the leading causes of cardiovascular mortality and morbidity [1]. Inflammation plays a pivotal role in driving the pathology of ACS, leading to the development of clinical trials targeting inflammation either acutely, such as in ASSAIL-MI, or chronically, such as in CANTOS in order to improve clinical outcomes. Alongside this, there has been extensive interest in utilising circulating inflammatory biomarkers for risk prediction post-ACS.

Hallmarks of the early inflammatory response to ACS are the exaggerated release of neutrophils from the bone marrow into circulation and infiltration of circulating neutrophils into the site of injury [2]. Neutrophils are effective phagocytes [3] and exert their antimicrobial and proinflammatory effects through the generation of reactive oxygen species (ROS), secretion of granular proteins and formation of neutrophil extracellular traps (NETs) [4]. In patients with myocardial infarction (MI), infiltrated neutrophils drive an inflammatory response at the site of infarction to facilitate the rapid clearance of necrotic cardiomyocytes and degradation of the surrounding extracellular matrix (ECM) [4, 5]. While these processes are necessary for successful scar deposition and myocardial healing post-MI, excessive neutrophil-driven inflammation has been associated with infarct expansion, maladaptive changes in left ventricular (LV) structure and function and, in turn, adverse outcomes [6].

The differential production and release of soluble granule contents are responsible for many of the phagocytic and oxidative functions of neutrophils in acute inflammation [3, 7]. Granule contents include myeloperoxidase (MPO) [4], serine proteases (proteinase 3 (PR3), neutrophil elastase (NE), and cathepsin G [4], azurocidin [8, 9], neutrophil gelatinase-associated lipocalin (NGAL) [10, 11], matrix metalloproteinases (MMP-8 and MMP-9) [4], calprotectin [12, 13], antimicrobial peptides (α-defensin) [14] and ficolins [15]. Previous studies have shown that these soluble factors are significantly elevated in the circulation of patients with ACS, and, in some instances, their release into circulation precedes the release of established markers of myocardial necrosis [16, 17]. The de novo process of NET formation [18, 19] is another important inflammatory function of neutrophils. NETs are abundantly present in coronary thrombi and have prothrombotic and proinflammatory roles during the development of atherosclerosis and in the acute inflammatory response to ACS [20].

Given the importance of neutrophils in driving a proinflammatory—sometimes excessive—response to ACS, the aim of this systematic review and meta-analysis was to assess the prognostic value of neutrophil-enriched soluble factors in predicting long-term major adverse cardiovascular events (MACE) in patients with ACS. Many circulating inflammatory mediators that are typically released during neutrophil degranulation can concurrently arise from other cellular sources. In this review, we examined markers that are known to be predominantly released by neutrophils including MPO, PR3, NGAL, calprotectin and markers of NETosis. We have termed these ‘neutrophil-enriched’ biomarkers. Other factors in which neutrophils are not considered the most significant source in blood circulation, including MMP-8, MMP-9, LL-37 and IL-8, were not assessed here.

## Methods

###  Systematic Review Search Strategy and Eligibility Criteria

This systematic review was conducted in accordance with PRISMA (Preferred Reporting Items for Systematic review and Meta-Analyses) and registered with PROSPERO (The International Prospective Register of Systematic Reviews; ID: CRD42021293391) [21, 22]. Circulating biomarkers which are described in prior literature to be predominantly, though not necessarily exclusively, released by neutrophils were considered ‘neutrophil-enriched’ in this study. These include double-stranded DNA (dsDNA), MPO-DNA, NE-DNA, citrullinated histone H3 (CitH3) (all of which are described as surrogate markers of NETosis), NE, MPO, NGAL, calprotectin, PR3, neutrophil α-defensin, azurocidin, cathepsin G, lactoferrin, ficolin and neutrophil-derived extracellular vesicles (EVs) (Supplementary Table 1). A comprehensive review of studies published from 1946 until October 2021 was conducted using the MEDLINE, EMBASE and EMBASE Classic, Scopus, SCIE (Web of Science) and Cochrane Central Register of Controlled Trials databases. Search terms are given in Supplementary Table 2. Duplicates were removed and additional studies were identified by manual searching of reference lists (Supplementary Table 3). Titles and abstracts were screened, and studies reporting the association of neutrophil-enriched biomarker levels with adverse outcome in patients with ACS were retrieved as full-text articles. Two independent reviewers (JY and AH) examined studies for eligibility using a standardised tool based on the PICOS format (Population, Intervention, Comparison, Outcomes, Studies) (Supplementary Figure 1). Studies were considered for inclusion according to the following criteria: the study population comprised ≥ 70% of patients with confirmed ACS; neutrophil-enriched biomarkers were sampled during hospital admission with ACS; and outcomes included, at minimum, all-cause mortality at ≥ 6 months following admission. Where disagreements arose, consensus was reached through discussion.

### Data Extraction and Quality Assessment

Data extraction and quality assessment of eligible studies were conducted following the recommendations of the Cochrane Review Group [23]. When required, authors were contacted to provide further clarification. Data were extracted regarding study design, inclusion criteria, patient characteristics, biomarker measurement (including inter-assay and intra-assay variability) and duration of follow-up. Categorical variables were reported as frequency (percentage), and continuous variables were given as median (interquartile range; IQR). For each reported MACE outcome, we extracted the odds ratio (OR), hazard ratio (HR), risk ratio (RR) or biomarker concentrations (median and IQR) and event rates, as applicable. Study quality (ranging from poor to high) was assessed using tools for cohort and case-control studies (Supplementary Figures 2 and 3), based on existing instruments [24–27]. Studies were excluded from further analysis if the overall quality rating was deemed poor due to lack of consideration of confounding variables in the study design and analysis, or if the minimum data required for meta-analysis was unable to be extracted.

###  Meta-analyses

We conducted meta-analyses of unadjusted summary statistics for neutrophil-enriched markers that were investigated in *n* > 3 studies. For studies reporting multiple MACE endpoints, only the outcome comprising the highest number of events was analysed. Different measures (RR, OR, HR) were used to report effect sizes across studies and required transformation prior to meta-analysis. For completeness of data, extracted unadjusted measures were transformed to unadjusted RR (95% CI). Crude event rates were compared across binary groupings of marker concentration (most commonly median) to estimate RR and concentrations given without sufficient event data were approximated to RR from standardised mean differences (Cohen’s *d*). For studies with rare outcomes (≤ 15%), OR were approximated to RR, while HR were directly pooled with RR. Pooled effect sizes with RR and 95% CI were calculated using the weighted inverse-variance method and restricted maximum likelihood (REML) estimations [28]. A random effects model with Hartung-Knapp adjustment was used to account for residual variability between studies [29, 30]. Heterogeneity was assessed with Cochran’s Q test (high heterogeneity was defined as *p* < 0.10) and the *I*^2^ test (high heterogeneity was defined as *I*^2^ > 75%) [29, 31, 32]. Potential publication bias was evaluated by assessment of funnel plot asymmetry with Egger’s regression test [33, 34]. All statistical tests were two-tailed, and significance was determined by *p* < 0.05. Statistical analyses were performed using R packages “meta”, “metafor” and “MetaUtility” in R Statistical Software version 4.1.2 (R Foundation for Statistical Computing, Vienna, Austria).

## Results

###  Study Characteristics

**Fig 1:**
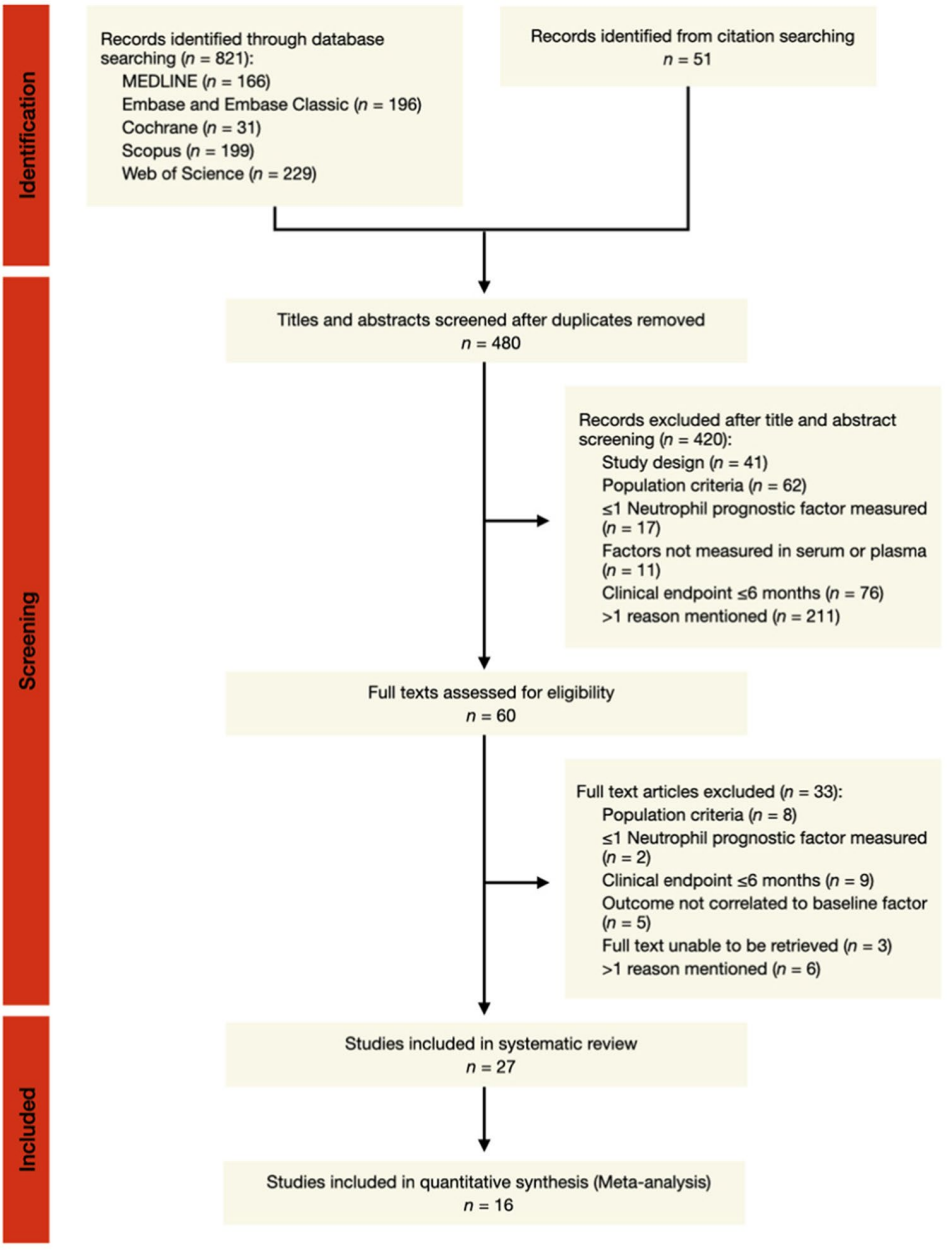
Overview of study selection for the systematic review and meta-analysis. The selection, screening, and inclusion of studies evaluating the association between neutrophil-enriched soluble markers and major cardiovascular events in ACS patients are detailed in the flow diagram, adapted from the PRISMA 2020 guidelines for systematic review reporting [6]

Of 872 articles identified, 27 studies (24 observational cohort and three case-control) were included in the systematic review (Fig. 1). A total of eight neutrophil-enriched soluble factors were measured in 17,831 patients with ACS. These factors were dsDNA, MPO-DNA, NE-DNA and CitH3—all surrogate markers of NETosis—as well as NGAL, MPO, calprotectin and PR3. The study characteristics are presented in Table 1. Patients were predominantly male (74 (67-79)%) with a median age of 62 (61-65) years. ACS encompasses a heterogenous population of STEMI, NSTEMI and unstable angina (UA) diagnoses. For this systematic review, five studies (19%) recruited patients with ACS, while 19 studies (70%) included patients with acute MI only (STEMI and/or NSTEMI); 11 of these studies specified a diagnosis of STEMI. Potential confounders such as renal dysfunction, inflammatory disease or cancer were excluded in a majority of the study populations (19 of 27 studies: 70%).

**Table 1:** Summary of the characteristics of the 27 studies included in the systematic review

Study (year), country	Marker	Sample size (MACE, %)	Age, years	Male, %	Sampling time from symptom onset	Inclusion criteria	Major exclusion criteria	Clinical endpoint	Follow-up
Akcay (2012), Turkey [50]^a^	NGAL	300 (19.8)	51.4 ± 6.1 (53 patients with NGAL > 46 ng/mL)	73.58	5 ± 2.2 hrs, before angiography	STEMI + PCI	Culprit lesion ≥ 50% stenosis^‡^, CABG, chronic inflammatory disease, infection or cancer	All-cause mortality and MACE^†^ (mortality, MI, HF, revascularisation)	1 year
Alfakry (2012), Finland [43]^a^	MPO	141 (29.1)	65.2 (IQR 10.2)	65.2	≤ 48 hrs, angiography NR	NSTEMI and UA ± PCI	Thrombolysis ≤ 48 hrs, coronary angioplasty ≤ 6 or CABG ≤ 3 months, chronic renal or hepatic disease or chronic antibiotic use	MACE^†^ (CV mortality, MI, UA, stroke)	Median 519 (138–924) days
Avci (2020), Turkey [47]^a^	NGAL	68 (20.6)	61.5 ± 14.7	82.4	4.6 ± 3 hrs, before angiography	STEMI ± PCI	Culprit lesion ≥ 50% stenosis^‡^, CABG or MI, LVEF < 55%, creatinine > 1.4 mg/dL, inflammatory disease, infection or cancer	CV death^†^	6 months
Baldus (2003), multicentre [68]^a^	MPO	547 (11.7)	61.7 ± 10.4	61	8.7 ± 4.9 hrs, before angiography	ACS with significant culprit lesion + PTCA	Prior MI, persistent ischemia, culprit lesion > 50% stenosis^‡^; recent surgery, GI bleeding ≤ 6 weeks; anticoagulant or thrombolytic agent; autoimmune disease or platelet count < 100 × 10^9^/L	All-cause mortality and MACE^†^ (CV mortality, MI, UA, stroke)	6 months
Barbarash (2017), Russia [54]^a^	NGAL	318 (88.4)	61.3 (95% CI 59.5–62.6)	72.3	12–14 days, before angiography	STEMI ± PCI	Prior PCI or CABG, autoimmune disease or cancer	MACE^†^ (CV mortality, MI, UA, stroke, acute HF)	3 years
Brügger-Andersen (2008), Norway [102]^a^	MPO	298 (27.8)	64 ± 13	79.3	4–6 days, after angiography	MI ± PCI	Severe HF (NYHA class IV), life expectancy < 2 years, late-stage cancer, GI bleeding, hepatic disease, thrombocytopenia or platelet count < 100 × 10^9^/L	MACE^†^ (CV mortality, ACS)	Median 45 months
Cavusoglu (2007), USA [69]^a^	MPO	182 (18.1)	64.8 ± 10	100	≥ 12 hrs from admission, before angiography	ACS ± PCI	GI bleeding	MACE^†^ (mortality, MI)	2 years
Hally (2021), New Zealand [41]^b^	MPO-DNA, NE-DNA, CitH3	300 (33)	68 (57–75)	67.7	3 (2–4) days, before angiography	MI ± PCI	Cardiac arrest, CHF, eGFR < 30, fibrinolytic agent ≤ 24 hrs, inflammatory disease, platelet function disorder or count < 100 × 10^9^/L	MACE^†^ (CV mortality, MI, stroke)	1 year
Helanova (2015), Czech Republic [51]^a^	NGAL	673 (6.4)	61 (46–78)	76.52	≤ 24 hrs, before angiography	STEMI + PCI	Cardiac arrest, inflammatory or connective tissue disease, cancer, life expectancy < 12 months or culprit lesion stenosis < 50%^‡^	All-cause mortality^†^	Median 2.7 years
Helseth (2019), Norway [37]^a^	dsDNA, MPO-DNA	251 (7.3)	60 (53–67)	82	Median 21.4 hrs, before angiography	STEMI + PCI	Prior MI, renal failure, contraindications to CMR or clinical instability	MACE^†^ (mortality, MI, stroke, HF, revascularisation)	1 year
Jensen (2010), Denmark [36]^b^	Calprotectin	141 (9.2)	68.6 ± 13.4 (13 patients who died at follow-up)	73.6	12.5 days (9.2–31.6) and 14.2 (IQR 9.6–21.5)^§§^, before angiography	STEMI with LAD occlusion + PCI	Lack of acute LAD occlusion, prior MI, HF, infection or inflammatory disease	All-cause mortality^†^	1 year
Kaya (2012), Turkey [70]^b^	MPO	73 (21.9)	56.5 ± 11.9	76.71	≤ 6 hrs, angiography NR	STEMI ± PCI	Valvular heart disease, chronic renal or hepatic disease inflammatory disease or cancer	MACE^†^ (mortality, MI, CHF, revascularisation, cerebrovascular event)	Mean 25 ± 16 months
Langseth (2020), Norway [38]^a^	dsDNA, MPO-DNA, CitH3	959 (19.9)	60.8 (range 24–94)	80	Median 24 hrs, after angiography	STEMI + PCI	Oral anticoagulant use	All-cause mortality or MACE^†^ (mortality, MI, revascularisation, HF, stroke)	Median 4.6 years
Lindberg (2012), Denmark [35]^a^	NGAL	584 (19)	66 ± 13; > 170.1 μg/L	73	3.2 (2.2–5.2) hrs, before angiography	STEMI + PCI	Troponin I increase ≤ 0.5g/L or no angiographic stenoses	All-cause mortality and MACE^†^ (CV mortality, MI, HF)	Median 23 (20-24) months
Liu (2021), China [48]^a^	NGAL	633 (6.5)	72.1 (68.2–79.2); ≥ 102.6 ng/L	52.92	≤ 24 hrs from admission, angiography NR	MI and stable angina ± PCI	Chronic renal or hepatic disease, severe aortic stenosis, cardiomyopathy, significant anaemia or cancer	CV death^†^	10 years
McCann (2009), multi (UK) [103]^a^	MPO	550 (9.8)	62 ± 13	70	6 (3.4–12.4) hrs, before angiography	Suspected ACS with chest pain ± PCI	Thrombolytic or anticoagulant use	All-cause mortality, non-fatal MI or MACE^†^ (mortality, MI)	1 year
Mocatta (2007), New Zealand [39]^a^	MPO	507 (15.4)	61.7 ± 11	80	24–96 hrs from admission, after angiography	MI ± PCI	Cardiogenic shock or in-hospital death ≤ 24 hours after onset	All-cause mortality^†^	5 years
Morrow (2008), multi [66]^b^	MPO	1524 (15.3)	61 (52–70)	66.99	≤ 24 hrs, before angiography	ACS with survival ≥180 days + tirofiban ± PCI	Prior PCI or CABG ≤ 6 months, persistent STE, LBBB, severe CHF, cardiogenic shock, systemic disease or creatinine > 2.5 mg/dL	Non-fatal ACS^†^	6 months
Ng (2011), UK [42]^a^	MPO, PR3	900 (22.8)	64.6 ± 12.4	70	2–5 days, after angiography	MI ± PCI	Cancer or recent surgery ≤ 1 month	All-cause mortality or hospitalisation with HF^†^	Mean 347 days
Nguyen (2019), France [44]^a^	NGAL	701 (11.5)	62.8 ± 14.4; without CI-AKI (88%)	74.61	On admission, before angiography	STEMI + PCI	Chronic haemodialysis or peritoneal dialysis	All-cause mortality	1 year
Nymo (2018), Sweden [52]^a^	NGAL	1121 (54.4)	69 (60–74); > 403 μg/L	70	Median 3 days, before angiography	MI ± PCI	Life expectancy < 1 year	All-cause mortality^†^	Median 167 months
Obeid (2020), multi (Switzerland ) [53]^a^	NGAL	1832 (10.5)	NR	79.15	≤ 72 hrs (91.3%), before angiography	MI ± PCI	No informed consent	All-cause mortality and MACE^†^ (mortality, MI, revascularisation, cerebrovascular events)	1 year
Peng (2019), China [49]^a^	NGAL	422 (32.2)	61 ± 13.1	74.17	≤ 24 hrs and 7 days, before angiography	NSTEMI with CTO + PCI	Rescue PCI or CABG, valvular heart disease or cardiomyopathy, CTO < 1 or > 1, life expectancy < 1 year, dialysis or contraindication to antiplatelet or anticoagulation therapy	MACE^†^ (CV mortality, stroke, revascularisation, cardiogenic shock)	2 years
Scirica (2010), multi [71]^a^	MPO	352 (6.8)	Mean 64	64.9	Median 22.4 hrs, before angiography	NSTEMI and UA ± PCI	Revascularisation, persistent STE, pulmonary oedema, systolic BP < 90 mmHg, cardiogenic shock, LBBB, LV hypertrophy, chronic hepatic disease, dialysis or life expectancy < 1 year	CV death, MI, HF	Mean 343 days
Wang (2018), China [55]^a^	dsDNA	142 (18.3)	59 (range, 28–88)	79.5	Mean 6.3 hrs, before angiography	STEMI + PCI	Valvular heart disease or cardiomyopathy, AF, chronic hepatic or renal disease, inflammatory or autoimmune disease or cancer	MACE^†^ (mortality, revascularisation, ACS, stroke)	Mean 24.5-25.71 months§
Wang (2019), China [56]^a^	Calprotectin	273 (17.2)	63.4 ± 8.5	62.4	On admission, before angiography	ACS with diabetes + PCI	Unsuccessful PCI (≤ TIMI grade 3), prior MI or CABG, infection or inflammatory disease, chronic hepatic or renal disease, long-term antiplatelet or anticoagulant use	MACE^†^ (CV mortality, MI, revascularisation)	1 year
Yndestad (2009), multi (Europe) [40]^a^	NGAL	236 (13.6)	67.4 ± 9.8	71.2	3 (1–7) days, angiography NR	MI with acute HF ± PCI	Planned revascularisation, systolic BP < 100 mmHg, ACEi or Ang II antagonist, UA, valvular heart disease	MACE^†^ (CV mortality, MI, stroke)	Median 2.7 years

Abbreviations: *ACEi*, angiotensin-converting enzyme inhibitors; *ACS*, acute coronary syndrome; *AF*, atrial fibrillation; *Ang II*, angiotensin II; *AP*, angina pectoris; *BP*, blood pressure; *CHF*, congestive heart failure; *CTO*, complete total occlusion; *CV*, cardiovascular; *GI*, gastrointestinal; *HF*, heart failure; *LAD*, left anterior descending artery; *LBBB*, left bundle-branch block; *LV*, left ventricular; *MI*, myocardial infraction; *NR*, not reported; *PTCA*, percutaneous transluminal coronary angioplasty, may also refer to PCI; *STE*, ST-segment elevation; *TIMI*, Thrombolysis In Myocardial Infarction (risk score for NSTEMI and UA); *UA*, unstable angina; *UK*, United Kingdom; *USA*, United States of America. ^a^Represents a cohort study; ^b^represents a case-control study; ^†^indicates the primary study endpoint; ^‡^refers to the percentage reduction of the intraluminal diameter of coronary arteries with stenosis; ^§^denotes the mean follow-up period in months for low and high dsDNA groups, respectively; ^§§^denotes the median time from symptom onset to sampling for patients who died and who survived at follow-up, respectively

In 24 studies (89%), blood samples were obtained within 3 days of symptom onset. Of these, patients in 17 studies (71%) were sampled within 24 h. Blood samples were taken prior to angiography in 18 studies (67%). Biomarkers were measured in plasma or serum samples in roughly equal proportions in 26 studies (46% and 54%, respectively), and only one study did not specify sample type. Nearly all studies measuring NGAL, MPO or NET-related biomarkers (dsDNA, MPO-DNA, NE-DNA and CitH3) used commercial or in-house developed ELISAs (72%, 90% and 100%, respectively) and other platforms included time-resolved immunofluorometric assays (TR-IFA). Notably, all eight studies using in-house developed assays for measuring biomarkers demonstrated intra-assay and inter-assay coefficients of variance of 3.8–10% and 0.63–14.8%, respectively [35–42].

The median length of follow-up was 1 (1–2.7) year from index admission, during which 16 (11-21) % of patients experienced MACE. Endpoints were variably defined among studies, and commonly included all-cause or cardiovascular death, non-fatal MI, stroke, repeat revascularisation and new-onset heart failure.

### Quality Assessment

The quality of the 24 cohort studies and the three case-control studies are summarised in Supplementary Table 4 and Supplementary Table 5, respectively. Three (12%) cohort studies were deemed of poor quality, while the remaining 24 studies were deemed of acceptable quality. A poor quality rating was given for three cohort studies due to potential confounders being inadequately addressed [40, 43] or for unclear outcome assessment methods [44]. Only one study was excluded from the final analysis for failure to adequately discuss the possible implications of inadequate adjustment for confounders and the inability to extract the minimum data required to create a risk ratio value [40]. Study objectives, inclusion criteria and methodology were generally well-defined and outcomes for primary endpoints were largely assessed using objective measures. Complete outcome data was provided for all participants in 22 studies, and participants who were reported as lost to follow-up or with incomplete data were generally excluded from analysis from the remaining papers. In total, 22 cohort studies sufficiently controlled for potential confounding factors in multivariate models. All three case-control studies addressed confounders either by sample matching or statistical adjustment based on clinical covariates.

###  Association of Neutrophil-Enriched Biomarkers and Cardiovascular Outcomes

#### NGAL

The relationship between NGAL and either mortality and/or MACE was examined in 11 cohort studies ranging in size from 68 to 1832 patients with follow-up ranging from 6 months to more than 13 years (Table 2). Most commonly, patients were categorised into high versus low NGAL groups based on medians, tertiles or quartiles (seven studies; 64%) or based on a threshold derived from ROC analysis (two studies, 18%). In three studies (27%), NGAL was treated as a continuous variable in statistical analysis (note that one study used both categorical and continuous approaches to analysis). A significant univariate association between higher levels of NGAL and adverse outcomes were reported in 10 of the 11 studies. In addition, statistically significant multivariate relationships were reported for NGAL and either mortality or MACE in eight (73%) of the 11 studies.

**Table 2:** Association between NGAL and MACE in patients with ACS

Study	Population	Reporting of effect	Endpoint and follow-up	Univariate analysis		Multivariate analysis	
				Unadjusted effect (95% CI)	*p*-value	Adjusted effect (95% CI)	*p*-value
Liu et al. (2021) [48]	*n* = 633 patients with MI and SA	Per 1 SD increase	CV death at 10 years^†^	HR 2.74 (1.75–5.37)	**< 0.001**	HR 2.62 (1.51–4.96)	**< 0.001**
Avci et al. (2020) [47]	*n* = 68 patients with STEMI	Grouped via median	CV death at 6 months^†^	357 (71–694) vs. 120 (9–513) ng/mL^‡^	**< 0.001**	*β*±SE 0.017 ± 0.007, (5.59)^2^	**0.01**
Obeid et al. (2020) [53]	*n* = 1832 patients with MI	Per 1 ng/mL increase	All-cause death^†^ and composite of death, MI, cerebrovascular events or revascularisation at 1 year^†^	OR 1.01 (1.007–1.013), all-cause death	**< 0.001**	HR 1.003 (0.999–1.008), all-cause death	0.16
				OR 1.006 (1.003–1.008), MACE	**< 0.001**	HR 1.001 (0.998–1.005), MACE	0.48
Nguyen et al. (2019) [44]	*n* = 701 STEMI patients treated with PCI	Grouped via highest vs. lowest tertile	All-cause death at 1 year^‡^	OR 2.4 (1.53–3.89)	**< 0.0001**	OR NR	NS
Peng et al. (2019) [49]	*n* = 422 NSTEMI patients with CTO	Grouped by ROC threshold (7-day NGAL)	Composite of CV death, cardiogenic shock, ischemic stroke or revascularisation at 2 years^†^	2.70 ± 1.11 vs. 2.21 ± 0.83 ng/mL^‡^	**< 0.001**	OR 2.01 (1.45–2.79)	**< 0.001**
Nymo et al. (2018) [52]	*n* = 1121 MI patients	Grouped by highest quartile vs. others	All-cause death during median 13.9 years^†^	HR 1.93 (1.63–2.28)	**< 0.001**	HR 1.63 (1.31–2.03)	**< 0.001**
Barbarash et al. (2017) [54]	*n* = 357 patients with STEMI	Grouped by lowest quartile vs. others	CV death^†^ and composite of CV death, MI, and hospitalisation due to UA, stroke or acute HF at 3 years^†^	2.36 vs. 1.61 ng/mL^‡^, CV mortality	**0.02**	OR NR, CV death	NS
				2.04 vs. 1.48 ng/mL^‡^, MACE	**0.01**	OR 2.9 (1.4–6.0), MACE	**0.003**
Helanova et al. (2015) [51]	*n* = 673 patients with STEMI	Grouped by ROC threshold	All-cause death at 1 year during median 2.7 years^†^	OR 1.939 (1.31–2.86)	**< 0.001**	OR 1.616 (1.027–2.543)	**0.038**
Akcay et al. (2012) [50]	*n* = 106 patients with STEMI undergoing PCI	^1^Per 1 ng/mL increase; ^2^grouped via median	All-cause death^†^ and composite of death, non-fatal MI, revascularisation or new CHF at 1 year^†^	HR 1.13 (1.08–1.25)^1^; 1.18 (1.09–1.37)^2^, all-cause death	**< 0.001**^**1**^**; < 0.01**^**2**^	HR 1.10 (1.06–1.22)^1^, all-cause death	**0.01**^**1**^
						HR 1.09 (1.04–1.19)^1^, MACE	**0.02**^**1**^
				HR 1.11 (1.04–1.24)^1^; 1.20 (1.12–1.34)^2^, MACE	**< 0.01**^**1**^**; < 0.01**^**2**^	HR 1.19 (1.06–1.22)^2^, all-cause death	**0.01**^**2**^
						HR 1.17 (1.08–1.27)^2^, MACE	**0.01**^**2**^
Lindberg et al. (2012) [35]	*n* = 584 patients with STEMI treated with PCI	Grouped by highest quartile vs. others	All-cause death^†^ and composite of CV death or hospitalisation due to MI or HF during median 23 (20–24) months^†^	Log rank^‡^, all-cause death	**< 0.001**	HR 2.00 (1.16–3.44), all-cause death	**0.01**
				Log rank^‡^, MACE	**< 0.001**	HR 1.51 (1–2.3), MACE	**0.05**
Yndestad et al. (2009) [40]	*n* = 236 MI patients with acute HF	Grouped via median	Composite of all-cause or CV death, MI or stroke during median 27 months^†^	Log rank^‡^	**< 0.001**	OR NR	**S**

Abbreviations: *ACEi*, angiotensin-converting enzyme inhibitor; *ARB*, angiotensin receptor blocker; *β ± SE*, beta coefficient ± standard error; *BMI*, body mass index; *BNP*, brain natriuretic protein; *BP*, blood pressure; *CAD*, coronary artery disease; *CRP*, C-reactive protein; *cTnI*, cardiac Troponin I; *cTnT*, cardiac Troponin T; *CV*, cardiovascular; *DM*, diabetes mellitus; *eGFR*, estimated glomerular filtration rate; *FBG*, fasting blood glucose; *GRACE*, Global Registry of Acute Coronary Events; *HF*, heart failure; *HFpEF*, HF with preserved ejection fraction; *HFrEF*, HF with reduced ejection fraction; *HR*, hazards ratio; *hsCRP*, high-sensitivity CRP; *LVEDD*, left ventricular end-diastolic diameter; *LVEF*, left ventricular ejection fraction; *MI*, myocardial infarction; *NGAL*, neutrophil gelatinase-associated lipocalin; *NR*, not reported; *NS*, not significant (*p* < 0.05); *OR*, odds ratio; *PCI*, percutaneous coronary intervention; *S*, *p*-value is statistically significant (unspecified); *SA*, stable angina; *95% CI*, 95% confidence interval; *TIMI*, Thrombolysis in Myocardial Infarction; *WBC*, white blood cell

^†^Indicates a primary endpoint; ^‡^indicates concentration is represented as median (IQR) or mean ± SD for cases versus controls, respectively; ^1^denotes continuous variables were assessed per unit change in concentration, as specified; ^2^denotes endpoints were compared in patients stratified according to pre-specified categories of biomarker concentration. Statistical significance was considered as two-tailed *p* ≤ 0.05 (bolded)

NGAL can also be released into the circulation by injured renal cells and may confound the neutrophil-mediated release of NGAL in ACS patients with renal impairment [45, 46]. To account for this, four of the 11 studies (36%) excluded patients with severe renal impairment as indicated by chronic renal disease, serum creatinine > 1.4 mg/dL, and/or requirement for haemodialysis [44, 47–49]. Though comparable exclusion criteria were not described in the remaining seven NGAL studies, five studies reported levels of serum creatinine (range 0.81–1.37 mg/dL) and eGFR (median range 65–117 mL/min/1.73 m^2^) within normal ranges [35, 40, 50–52], and only a small proportion of patients were reported as requiring dialysis in three studies (range 0.3–2.0%) [35, 50, 53]. In contrast, Barbarash et al. did not state whether the moderate proportion of patients in this study (29%) with marked renal impairment (eGFR < 60 mL/min/1.73 m^2^) were accounted for in the analysis [54].

#### MPO

The prognostic ability of circulating MPO was assessed in 10 studies (eight cohorts and two case-control) that included 9074 patients with ACS (Table 3). Three studies (30%) treated MPO as a continuous variable, while eight (80%) studies grouped patients into high versus low MPO levels (one study did both). A total of seven studies reported an association between increased levels of MPO and adverse clinical outcome on univariate analysis, with statistical significance retained in six studies.

**Table 3:** Association between MPO and MACE in patients with ACS

Study	Population	Reporting of effect	Endpoint and follow-up	Univariate analysis		Multivariate analysis	
				Unadjusted effect (95% CI)	*p*-value	Adjusted effect (95% CI)	*p*-value
Alfakry (2012) [43]	*n* = 141 patients with acute non-Q-wave infarction and UA	Highest quartile of MPO concentration vs. lowest quartiles	Composite of CV mortality, MI, UA or ischaemic stroke at 1 year^†^	275.3 (238.4) vs. 160.8 (241.0) ng/mL^‡^	**0.017**	RR 3.540 (1.600–7.831)	**0.002**
Ng (2011) [42]	*n* = 900 patients with MI	Treated as continuous variable	All-cause death^†^, hospitalisation due to HF^§†^ and combined at 1 year^†^	OR NR^1^, MACE	NS	HR NR^‡^, MACE	NS
Scirica (2010) [71]	*n* = 4352 patients with NSTEMI and UA	Grouped by ROC threshold	Composite of CV death and HF^†^, CV death^†^, recurrent MI^†^ and hospitalisation due to HF at 1 year^†^	No univariate analysis		HR 1.49 (1.18–1.88), MACE	**0.001**
						HR 1.49 (1.12–1.97), CV death	**0.006**
McCann (2009) [103]	*n* = 550 patients with chest pain	Grouped by highest quartile vs. others	Composite of all-cause death or MI at 1 year^†^	OR 0.8 (0.4–1.6)	0.51	No multivariate analysis	
Brügger-Andersen (2008) [102]	*n* = 298 MI patients	Grouped by highest quartile vs. others	Recurrent ACS or CV death during median 45 months^†^	HR NR	**S**	HR 1.04 (0.59−1.86)	0.89
Morrow (2008) [66]	*n* = 1524 patients with MI and UA surviving to 180 days	^1^Treated as continuous variable; ^2^grouped via median	Composite of recurrent MI or hospitalisation due to ACS at 6 months^†^	Log rank^2^	0.14	OR 1.15 (0.99–1.34)^1^; 1.26 (0.95–1.68)^2^	0.072^1^; 0.072^2^
Cavusoglu (2007) [66]	*n* = 182 patients with MI and UA with coronary angiography	Treated as continuous variable	MI-free survival at 2 years^†^	OR 1.51 (1.05–2.18)	**0.028**	OR 1.6 (1.09–2.36)	**0.017**
Mocatta (2007) [39]	*n* = 507 patients with MI	Grouped via median	All-cause death at 5 years^†^	Log rank^2^	**0.0011**	RR 1.8 (1.1–3.1)	**0.03**
Baldus (2003) [68]	*n* = 547 ACS patients treated with PCI	Grouped by highest tertile vs. others	All-cause death^†^, composite of CV death, MI, UA, or ischaemic stroke^†^ and combined death and MI at 6 months^†^	HR NR, mortality and MI	**0.012**	HR 2.25 (1.32–3.82), death and MI	**0.003**
						HR 2.11 (1.21–3.67), death and MI	**0.008**

Abbreviations: *ASA*, acetylsalicylic acid; *BMI*, body mass index; *BNP*, brain natriuretic protein; *BP*, blood pressure; *CABG*, coronary artery bypass graft; *CAD*, coronary artery disease; *CD40L*, CD40 ligand; *CRP*, C-reactive protein; *cTnI*, cardiac Troponin I; *cTnT*, cardiac Troponin T; *CV*, cardiovascular; *DM*, diabetes mellitus; *eGFR*, estimated glomerular filtration rate; *FBG*, fasting blood glucose; *GRACE*, Global Registry of Acute Coronary Events; *HF*, heart failure; *HFpEF*, HF with preserved ejection fraction; *HFrEF*, HF with reduced ejection fraction; *HR*, hazards ratio; *hsCRP*, high-sensitivity CRP; *LVEDD*, left ventricular end-diastolic diameter; *LVEF*, left ventricular ejection fraction; *MI*, myocardial infarction; *NGAL*, neutrophil gelatinase-associated lipocalin; *NR*, not reported; *NS*, not significant (*p* < 0.05); *OR*, odds ratio; *PCI*, percutaneous coronary intervention; *S*, *p-*value is statistically significant (unspecified); *SA*, stable angina; *95% CI*, 95% confidence interval; *TIMI*, Thrombolysis in Myocardial Infarction; *VEGF*, x; *WBC*, white blood cell. ^†^Indicates a primary endpoint; ^‡^indicates concentration is represented as median (IQR) or mean ± SD for cases vs. controls, respectively; ^§^HF requiring high-dose diuretics, inotropes or intravenous nitrate; ^§§^indicates statistical adjustment for variable identified as univariate predictors of MACE; ^1^denotes continuous variables were assessed per unit change in concentration, as specified; ^2^denotes endpoints were compared in patients stratified according to pre-specified categories of biomarker concentration. Statistical significance was considered as two-tailed *p* ≤ 0.05 (bolded)

#### Markers of NETosis

The association between markers of NETosis (dsDNA, MPO-DNA, NE-DNA and CitH3) and MACE were examined in 1649 patients across four studies (Table 4). Increased levels of dsDNA were independently predictive of MACE in three studies [37, 38, 55], two of which were conducted by the same institution [37, 38]. Neither MPO-DNA, NE-DNA nor CitH3 were found to predict MACE after ACS in three studies [37, 38, 41].

**Table 4:** Association between NETosis components and MACE in patients with ACS

Study	Population	Reporting of effect	Endpoint and follow-up	Univariate analysis		Multivariate analysis	
				Unadjusted effect (95% CI)	*p*-value	Adjusted effect (95% CI)	*p*-value
dsDNA							
Langseth et al. (2020) [38]	*n* = 956 patients with STEMI	^2^ Grouped by median ^3^Grouped by highest quartile vs. others	All-cause death^†^ and composite of death, MI, stroke, hospitalisation due to HF, or revascularisation > 3 months at median 4.6 years^†^	HR 3.36 (1.95–5.78)^3^; log rank^2^; 460 (407–508) vs. 411 (370–466) ng/mL^‡^, all-cause death	**< 0.001**^3^; **< 0.001**^2^; **< 0.001**^‡^	HR 2.28 (1.19–4.36)	**0.013**
				Log rank^2^; 429 (317–481) vs. 412 (372–466) ng/mL^‡^, MACE	0.052^2^; 0.255^‡^	HR 2.06 (1.08-3.93)	**0.029**
Helseth et al. (2019) [37]	*n* = 251 STEMI patients treated with PCI	Grouped by median	Composite of all-cause mortality, MI, revascularisation ≥3 months, stroke, or hospitalisation due to HF at 1 year^†^	HR 5.9 (1.7–20.3)	**0.005**	HR 6.7 (1.9–23.2)	**0.003**
Wang et al. (2018) [55]	*n* = 142 STEMI patients treated with PCI	Grouped by ROC threshold	All-cause^†^ and CV death^†^ & composite of death, ACS, PCI or CABG, or stroke at mean 24.5 months^†^	Log rank^2^, MACE	**0.04**	OR 7.43 (1.25–4.07), MACE	**0.027**
MPO-DNA							
Hally et al. (2021) [41]	*n* = 100 patients with MI	^2^ Grouped by median ^§^Median z-score ^§§^Median z-score with sP-selectin	Composite of CV death, non-fatal MI, or ischemic stroke at 1 year^†^	OR 1.13 (0.7–1.83)^2^; OR 1.28 (0.72–2.1)^§^; OR 1.86 (1.13–3.08)^§§^	0.62; 0.33^§^; **0.015**^§§^	No multivariate analysis	
						OR 1.94 (1.16–3.25)^§§^	**0.011**
Langseth et al. (2020) [38]	*n* = 956 patients with STEMI	^2^Grouped by median	*See Langseth et al. (2020)*	Log rank^2^; 0.167 (0.14–0.25) vs. 0.18 (0.14–0.26) OD^‡^, all-cause death	0.36^2^; 0.91^‡^	No multivariate analysis	
				Log rank^2^; 0.17 (0.14–0.24) vs. 0.18 (0.14–0.27) OD^‡^, MACE	0.16^2^; 0.33^‡^		
Helseth et al. (2019) [37]	*n* = 251 STEMI patients treated with PCI	Grouped by median	*See Helseth et al. (2019)*	HR NR	NS	No multivariate analysis	
**NE-DNA**							
Hally et al. (2021) [41]	*n* = 100 patients with MI	Grouped by median	*See Hally et al. (2021)*	OR 1.06 (0.66–1.72)	0.81	No multivariate analysis	
Citrullinated histone 3							
Hally et al. (2021) [41]	*n* = 100 patients with MI	Grouped by median	*See Hally et al. (2021)*	OR 1.43 (0.89–2.33)	0.14	No multivariate analysis	
Langseth et al. (2020) [38]	*n* = 956 patients with STEMI	^2^ Grouped by median	*See Langseth et al. (2020)*	Log rank^2^; 10.25 (4.96–17.32) vs. 9.07 (4.83–17.24) ng/mL^‡^, all-cause death	0.092^2^; 0.6^‡^	No multivariate analysis	
				Log rank^2^; 92 (4.48–16.52) vs. 9.32 (4.91–17.30) ng/mL^‡^, MACE	0.88^2^; 0.46^‡^		

Abbreviations: *BMI*, body mass index; *BNP*, brain natriuretic protein; *BP*, blood pressure; *CABG*, coronary artery bypass graft; *CAD*, coronary artery disease; *CitH3*, citrullinated histone H3; *H3(cit)*, see CitH3; *CRP*, C-reactive protein; *cTnI*, cardiac Troponin I; *cTnT*, cardiac Troponin T; *CV*, cardiovascular; *DM*, diabetes mellitus; *eGFR*, estimated glomerular filtration rate; *FBG*, fasting blood glucose; *GOT*, glutamic oxalacetic transaminase; *GRACE*, Global Registry of Acute Coronary Events; *HF*, heart failure; *HFpEF*, HF with preserved ejection fraction; *HFrEF*, HF with reduced ejection fraction; *HR*, hazard ratio; *hsCRP*, high-sensitivity CRP; *LVEDD*, left ventricular end-diastolic diameter; *LVEF*, left ventricular ejection fraction; *MI*, myocardial infarction; *NE*, neutrophil elastase; *NGAL*, neutrophil gelatinase-associated lipocalin; *NR*, not reported; *NS*, not significant (*p* < 0.05); *OR*, odds ratio; *PCI*, percutaneous coronary intervention; *S*, *p*-value is statistically significant (unspecified); *SA*, stable angina; *95% CI*, 95% confidence interval; *TIMI*, Thrombolysis in Myocardial Infarction; *WBC*, white blood cell. ^†^Indicates a primary endpoint; ^‡^indicates concentration is represented as median (IQR) or mean ± SD for cases vs. controls, respectively; ^1^denotes continuous variables assessed per unit change in concentration, as specified; ^2^denotes endpoints compared in patients stratified according to median biomarker concentration; ^3^denotes endpoints compared in patients stratified according to pre-specified categories of biomarker concentration. ^§^Denotes effect size was calculated using the composite NET z-score (comprising z-scores of MPO-DNA% of NET standard, NE-DNA% of pooled serum standard and H3 (cit)% of NET standard) with platelet count; ^§§^denotes effect parameter was calculated using composite NET z-score with platelet count *and* sP-selectin. Statistical significance was considered as two-tailed *p* ≤ 0.05 (bolded)

#### Calprotectin

Adjusted HRs were extracted from two studies evaluating the prognostic significance of calprotectin in 207 patients with ACS [36, 56] (Table 5). All-cause mortality was reported in 9.2% of patients in Jensen et al. [36]and in 17.2% of patients in Wang et al [56] within the first year after coronary revascularisation. Both studies observed a statistically significant independent association between elevated calprotectin levels and MACE.

**Table 5:** Association between calprotectin and proteinase-3 and MACE in patients with ACS

Study	Population	Reporting of effect	Endpoint and follow-up	Univariate analysis		Multivariate analysis	
				Unadjusted effect (95% CI)	*p*-value	Adjusted effect (95% CI)	*p*-value
Calprotectin							
Wang et al. (2019) [56]	*n* = 273 ACS patients with diabetes treated with PCI	Grouped by ROC threshold	Composite of CV death, non-fatal MI or unplanned revascularisation at 1 year^†^	HR 1.56 (1.08–4.62)	**0.01**	HR 2.11 (1.14–6.65)	**< 0.01**
Jensen et al. (2010) [36]	*n* = 141 STEMI patients with acute LAD occlusion	^1^Continuous variable; ^2^ Grouped by ROC threshold	All-cause death at 1 year^†^	HR 1.30 (1.1–1.5)^1^; 6.28 (0.4–28.1)^2^	**< 0.001**^**1**^**; 0.02**	HR 1.28 (1.1–1.5)^1^, 7.28 (1.6–32.9)^2^	**< 0.001**^**1**^**; 0.01**
Proteinase-3							
Ng et al. (2011) [42]	*n* = 900 patients with MI	^1^Continuous variable ^2^Grouped by median	All-cause death, hospitalisation due to HF^§^, and composite of death and HF at 1 year^†^	OR 6.42 (2.25–18.3)^1^; NR^2^	**0.001**^**1**^**; < 0.001**^2^	HR 3.80 (1.78–8.14)^2^	**0.001**^**2**^

Abbreviations: *ACEi*, angiotensin-converting enzyme inhibitors; *BMI*, body mass index; *BNP*, brain natriuretic protein; *BP*, blood pressure; *CABG*, coronary artery bypass graft; *CAD*, coronary artery disease; *CitH3*, citrullinated histone H3; *H3(cit)*, see CitH3; *CRP*, C-reactive protein; *cTnI*, cardiac Troponin I; *cTnT*, cardiac Troponin T; *CV*, cardiovascular; *DM*, diabetes mellitus; *eGFR*, estimated glomerular filtration rate; *FBG*, fasting blood glucose; *GOT*, glutamic oxalacetic transaminase; *GRACE*, Global Registry of Acute Coronary Events; *HF*, heart failure; *HR*, hazard ratio; *LVEF*, left ventricular ejection fraction; *MI*, myocardial infarction; *NR*, not reported; *NS*, not significant (*p* < 0.05); *OR*, odds ratio; *PCI*, percutaneous coronary intervention; *S*, *p-*value is statistically significant (unspecified); *SA*, stable angina; *95% CI*, 95% confidence interval; *TIMI*, Thrombolysis in Myocardial Infarction; *WBC*, white blood cell. ^†^Indicates a primary endpoint; ^‡^indicates concentration is represented as median (IQR) or mean ± SD for cases vs. controls, respectively; ^§^HF requiring high-dose diuretics, inotropes or intravenous nitrate. ^1^Denotes continuous variables assessed per unit change in concentration, as specified; ^2^denotes endpoints compared in patients stratified according to pre-specified categories of biomarker concentration. Statistical significance was considered as two-tailed *p* ≤ 0.05 (bolded)

#### PR3

Ng et al. [42] demonstrated a significant independent association between MACE at 1 year and PR3 (per 10-fold increase in log concentration) in 900 patients with ACS (Table 5). MACE was defined as all-cause mortality, recurrent MI and hospitalisation with HF and was reported in 16% of patients.

### Meta-analysis of NGAL

**Fig. 2:**
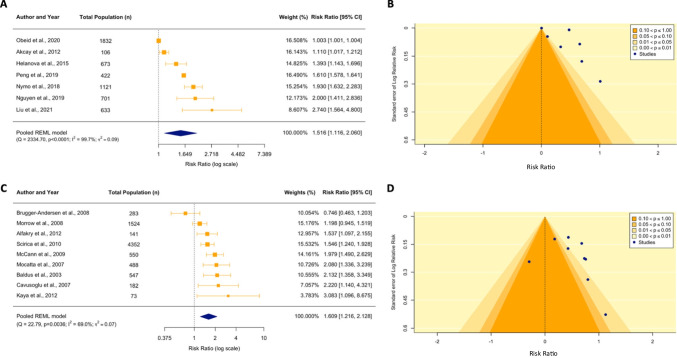
Meta-analysis for neutrophil gelatinase-associate lipocalin (NGAL) and myeloperoxidase (MPO). **A**, **C** The forest plots illustrate the unadjusted individual and summary risk ratios (RR) for MACE among ACS patients with high and low baseline levels of NGAL (**A**) and MPO (**C**) during hospital admission. The summary RR is indicated by the diamond and was calculated based on random effects meta-analysis using REML estimation and Hartung-Knapp adjustment. Weightings for each of the studies included in the model are proportionally reflected by the size of the box. The widths of the intersecting horizontal lines indicate the 95% confidence intervals. **B**, **D** Evidence used for publication bias assessment for NGAL (**B**) and MPO (**D**) is illustrated in the funnel plots, which present the logRR of studies investigating either NGAL or MPO against the inverse standard error. The shaded contours represent varying levels of statistical significance as indicated by the key. The null effect is denoted by the vertical dotted line

Of the 11 studies investigating circulating NGAL levels, risk ratio data from 7 studies (64%) was extracted and pooled in a random effects model [44, 48–53]. Within these studies, a total of 1384 MACE events were recorded for 5488 patients with ACS. Four studies were excluded from meta-analysis due to incomplete study data. Composite MACE [49, 50, 53] and all-cause mortality endpoints were each assessed in three studies [44, 51, 52], while CV mortality was assessed in one study [48]. Meta-analysis demonstrates that, when dichotomized by high or low baseline NGAL levels, high levels were associated with a 51.6% increased risk of MACE (unadjusted RR 1.52, 95% CI 1.16–2.06, *p* = 0.016) compared to low levels (used as the reference) (Fig. 2A). There was evidence of substantial statistical heterogeneity in the model, with *τ*^2^ of 0.09 (95% CI 0.03–0.57, *p* < 0.0001) and *I*^2^ of 99.7% (95% CI 99.1–100.0%). Observation of a non-uniform distribution of study *p*-values in the contour funnel plot alongside a significant Egger’s test (*z* = 2.65, *p* = 0.04) indicates likely publication bias resulting from small study effects (Fig. 2B).

### Meta-analysis of MPO

Of the 10 studies describing the association of baseline MPO levels with MACE, risk ratio data was extracted from nine studies and pooled in a random effects meta-analysis. Study effect estimates could not be approximated to RR for Ng et al. due to incomplete study data [42]. A total of 1577 MACE events were captured in 8174 patients with ACS, ranging from 6 months to 5 years after index admission. Elevated circulating levels of MPO were significantly associated with an increased risk of MACE (unadjusted RR 1.61, 95% CI 1.22–2.13, *p* = 0.004) compared to low MPO levels (Fig. 2C). Moderate heterogeneity was noted in the model (*τ*^2^ 0.07, 95% CI 0.01–0.52; *p* = 0.004 and *I*^2^ 70.0%, 95% CI 25.8–94.1). The funnel plot for MPO indicates the presence of asymmetry with a non-uniform distribution of *p*-values for the studies in the contour funnel plot; however, Egger’s test was non-significant suggesting small study effects are not driving publication bias (*z* = 1.11, *p* = 0.3) (Fig. 2D).

## Discussion

We identified 27 studies investigating the prognostic association of either NGAL, MPO, calprotectin, PR3 or markers of NETosis (dsDNA, MPO-DNA, NE-DNA, CitH3) with MACE in patients with ACS. NGAL was the most studied marker, with 11 studies reporting associations between NGAL and MACE. Ten of these found higher levels of NGAL were associated with worse outcomes. Seven studies contained sufficient information to be incorporated into a random effects meta-analysis, which showed that increased levels of circulating NGAL during hospital admission were associated with a 52% increase in risk for long-term MACE. MPO was investigated in 10 studies, 9 of which could be incorporated into a meta-analysis. This analysis demonstrated that increased MPO levels at presentation with ACS were associated with a 61% increased risk of long-term MACE. The other markers were less well studied, but there was some evidence of an association between increased circulating levels of calprotectin, PR3 and dsDNA (used as a surrogate marker of NETosis) and long-term MACE.

Both the multivariate outcomes reported within the individual studies, and the meta-analysis of the unadjusted outcomes support the view that elevated levels of NGAL are associated with worse clinical outcomes. NGAL is an acute-phase glycoprotein contained within specific granules of neutrophils and elicits antimicrobial and chemotactic functions at sites of inflammation [51, 52, 57]. Within the post-ischemia setting, NGAL acts to enhance MMP-9 activation by forming the stable NGAL/MMP-9 complex which, in turn, can amplify and prolong ECM degradation during infarct remodelling [57]. Within the coronary and systemic circulation, neutrophils are the principal source of circulating NGAL [10, 11]. However, NGAL is also an early biomarker of acute kidney injury as a result of secretion from renal tubular cells [58, 59]. It is possible that the release of NGAL by injured renal cells in ACS patients that also have poor kidney function may confound the prognostic association of neutrophil-derived NGAL [40].

Eight of the studies included in this systematic review noted that increased levels of NGAL at baseline may reflect pre-existing renal dysfunction in addition to neutrophil activation during acute inflammation [35, 40, 44, 47, 48, 50, 52, 53]. Due to this interaction [60–63], it is important to consider the ways in which potential confounding effects of renal-mediated NGAL. Chronic kidney disease was an exclusion criterion in four of 11 studies (37%) [44, 47–49]. In addition, all but two study [40, 47] outcomes were adjusted for creatinine or eGFR in multivariate analysis. Most of the studies (8 of 11; 72%) measured NGAL levels in pre-angiography blood samples prior to any potential renal injury caused by the administration of contrast [35, 40, 44, 47, 50–53]. One study did not address renal function at all [40].

MPO is a haemoprotein released from the azurophilic granules of mature neutrophils and is also released, to a lesser degree, by other immune cells such as monocytes and tissue-associated macrophages [64]. Within atherosclerotic lesions, MPO plays a prominent role in plaque destabilisation [65]. The oxidation of ROS substrates such as nitric oxide (NO) and protein and lipid components in the vascular endothelium serves as important mechanisms of MPO-mediated endothelial dysfunction [66]. As an early participant in acute inflammation, MPO induces proteolytic changes in tissue mediators such as MMPs and plasminogen activator inhibitor-1 (PAI-1) to promote ECM degradation in the infarct [67]. Seven out of 10 studies (70%) reported a significant univariate association between increased circulating levels of MPO and long-term MACE, in which MPO remained an independent predictor of MACE in six studies [39, 43, 68–71]. Meta-analysis of the unadjusted outcomes supports this association between higher levels of MPO and increased risk.

NETs are extracellular scaffolds composed of decondensed chromatin, citrullinated histones and granular proteins such as MPO and NE [72–76]. Beyond their antimicrobial role, NETs accumulate in coronary thrombi in ACS to exert prothrombotic functions [77, 78]. For example, NETs act to promote fibrin deposition and thrombin generation through the activation of tissue factors and platelet aggregation [79, 80]. Much of the literature investigating NETs as novel predictors of cardiovascular risk in ACS has focused on circulating dsDNA as a surrogate marker of NETosis [20, 38]. In this review, increased levels of circulating dsDNA in MI patients were found to be a significant predictor of MACE in three studies [37, 38, 55]. A caveat in ACS patients is that dsDNA is not specific to NETosis but can also be released from dying cardiomyocytes. Other surrogate NET markers (namely, MPO-DNA, NE-DNA and CitH3) have greater specificity for NETosis than dsDNA [81–83]. Yet, based on the studies identified in this review, there was no evidence supporting their individual utility for predicting MACE [37, 38, 41].

We found less literature on PR3 and calprotectin. Like MPO, PR3 is a neutrophil-derived serine protease [84]. PR3 has been associated with promoting neutrophil recruitment through its ability to activate certain chemokines and cytokines [85, 86]. In Ng et al., increased plasma levels of a PR3 complex were significantly associated with long-term risk of MACE post-MI [42]. Calprotectin is predominantly released by neutrophils [12, 17, 87, 88] and is involved in a myriad of inflammatory functions including phagocytosis [89], neutrophil and monocyte recruitment [90, 91] and cytokine and chemokine production [92]. Both Wang et al. [56] and Jensen et al. [36] reported calprotectin was associated with long-term MACE after adjustment for confounding variables.

Publication bias is an issue to be mindful of when interpreting the results from any meta-analysis. In our meta-analyses of NGAL and MPO, we observed some clustering of the studies in the *p* = 0.01–0.05 significance bands in the contour funnel plots. Furthermore, a significant Egger’s test result for NGAL suggests that publication bias due to small study effects is likely to be present. Therefore, it is possible that studies are more likely to be published if reporting a significant association between NGAL and MACE. In the case of MPO, the presence of publication bias is not as clear cut. Despite the presence of asymmetry in the standard funnel plot and the observation of a non-uniform distribution of *p*-values in the contour funnel plot, the Egger’s test was not significant. An Egger’s test is often used as an objective measure of asymmetry for funnel plots and is useful for assessing the risk of small study bias inside a meta-analysis, reflecting the fact that greater variance is often observed in smaller studies [34]. However, publication bias should only be one factor when considering the presence of asymmetry in a funnel plot. Other important sources of selection bias include outcome reporting bias, clinical heterogeneity and poor methodological design, all of which are often associated with smaller studies [93]. It is difficult to ascertain the definitive source of bias that may exist in our forest plots for the NGAL and MPO studies, although we present some plausible reasoning in our limitations. It must be highlighted that these meta-analyses were conducted on 7 and 9 studies respectively, which is below the minimum number of 10 studies recommended for analysing publication bias [93]. Therefore, the results of bias must be interpreted cautiously from our study as the Egger’s test may not be powered to distinguish real asymmetry from chance. We would like to highlight that small studies, or large effect sizes reported by small studies, are not problematic in themselves. There is valuable information to be gathered from these types of studies. However, it is the selective publication of results which favours the publication of smaller studies more commonly than from larger studies that then causes bias to become an issue.

### Limitations

These studies discussed in this review are cohort and case-control designs, none of which constitute high-quality evidence for evaluating the prognostic utility of the biomarkers studied. In addition, for the most studied biomarkers, NGAL and MPO, there is evidence that publication bias may contribute to the effect observed in both meta-analyses.

The heterogeneity of timing of blood sampling among studies included within this review is a potential confounder. The exact timing of peak neutrophil activity is unclear in humans [4, 94–97]. There is currently no consensus regarding an optimal timepoint to measure biomarkers in the acute stage of myocardial infarction, nor whether biomarkers measured at a single moment in time can sufficiently capture their contribution to MACE risk. Studies examining biomarkers at multiple timepoints may provide insight into the impact of timing on prognostic utility.

Length of follow-up and definitions of MACE varied between studies, contributing to the variance in MACE rates observed (from 6.4 [51] to 88.4% [54]). In Barbarash et al. [54], the high MACE rate of 88.4% may likely reflect the higher baseline risk of the population as well as the inclusion of unstable angina as a MACE endpoint. The variance in MACE rates, as well as differences in study populations and length of follow-up, are likely contributors to the high heterogeneity observed in meta-analysis of MPO and NGAL.

For clinically practical reasons, the studies included here examined biomarkers exclusively in peripheral blood samples. However, soluble mediators may exhibit biological compartmentalisation that cannot be captured in peripheral samples. Previous studies have revealed a significantly higher expression of markers of neutrophil degranulation and NETosis in coronary thrombi than in peripheral plasma [98], and in infarct-related arteries, but not in samples taken from non-infarct-related coronaries [79, 99]. By contrast, peripheral and coronary dsDNA are reported to be highly intercorrelated [55]. It is possible that, compared to peripheral blood, coronary sinus blood may offer a better reflection of these biomarkers within the ischaemic microenvironment. However, questions concerning the clinical practicality, safety and invasiveness of coronary sinus sampling remain.

It must be noted that this systematic review and meta-analyses focused on studies reporting a correlation between neutrophil biomarkers and clinical outcome in ACS patients. These studies did not investigate a causative association between the investigated biomarker and MACE, such that interpretation of these relationships must be viewed with caution when considering what is driving adverse outcomes in these patients. However, clinical trials such as CANTOS [100] and ASSAIL-MI [101] have presented compelling results to suggest that the level of inflammation in patients with cardiovascular disease is linked with cardiac outcomes. Both CANTOS and ASSAIL-MI show that targeting specific inflammatory cytokines with a monoclonal antibody therapy can reduce cardiovascular events (CANTOS) and limit infarct expansion (ASSAIL-MI). These results give rise to a plausible mechanistic connection between the burden of inflammation during the acute phase of an MI and MACE at 1 year. Whether neutrophil biomarkers, like the ones discussed in this study, are involved in this mechanistic process remains unclear.

## Conclusion

This systematic review and meta-analysis examined the prognostic utility of eight neutrophil-enriched biomarkers in patients with ACS. In a meta-analysis, increased levels of circulating NGAL and MPO were found to be significantly associated with long-term adverse cardiovascular outcomes in patients with ACS, supporting the possibility that neutrophil-mediated inflammation may play an important role in myocardial injury processes. For the remaining markers, promising data indicates the association of dsDNA, calprotectin and PR3 with long-term MACE post-ACS. However, no such association was found for MPO-DNA, NE-DNA or CitH3 (all surrogate markers of NETosis). While these eight circulating biomarkers are predominantly produced by neutrophils, the release of some of these (dsDNA and NGAL) are likely to be confounded by other physiological processes in patients with ACS (cardiomyocyte necrosis and renal injury, respectively).

### Supplementary Information

ESM 1 (DOCX 39312 kb)

## Declarations

No human studies were carried out by the authors for this article.
