# Supplementary material for: Neutrophil-Enriched Biomarkers and Long-Term Prognosis in Acute Coronary Syndrome: a Systematic Review and Meta-analysis
Source: J Cardiovasc Transl Res. 2023 Aug 18;17(2):426–47. doi: 10.1007/s12265-023-10425-2 (PMC11052791; doi:10.1007/s12265-023-10425-2)
Supplement: Supplementary file 1 — (DOCX 39312 kb) [file 12265_2023_10425_MOESM1_ESM.docx]

Neutrophil-enriched biomarkers and long-term prognosis in Acute Coronary Syndrome: a systematic review and meta-analysis

Jacquelina Yiu

Kathryn E Hally, ORCID ID: 0000-0003-3608-3795

Peter D Larsen, ORCID ID: 0000-0001-8706-9032

Ana S Holley, ORCID ID: 0000-0003-0469-134X

Wellington Cardiovascular Research Group

Department of Surgery and Anaesthesia

University of Otago, Wellington

PO Box 7343

Wellington South

New Zealand

Supplementary material

Supplementary Table 1. Candidate markers commonly released by neutrophils in inflammation – activation, function, and inclusion in review.

Supplementary Table 2. Search strategy for all databases.

Supplementary Table 3. Supplementary search strategy.

Supplementary Table 4. Quality assessment for included cohort studies.

Supplementary Table 5. Quality assessment of case-control studies.

Supplementary Figure 1. Eligibility screen tool.

Supplementary Figure 2. Quality assessment tool for cohort studies.

Supplementary Figure 3. Quality assessment tool for case-control studies.

Supplementry Figure 4. Standard funnel plots.

Supplementary Table 1. Candidate markers commonly released by neutrophils in inflammation – Activation, function, and inclusion in review. This table summarises the role and cellular source of soluble factors commonly released by activated neutrophils under inflammatory conditions. Markers excluded from inclusion in the systematic review are noted.

| Marker | Action in acute inflammation | Presence in neutrophils | Released from other cells | Included in the review |
| --- | --- | --- | --- | --- |
| Azurophilic (primary) granules | | | | |
| Neutrophil elastase (NE) | Proinflammatory serine protease – acts as a monocyte chemoattractant, involved in NET formation (1), platelet aggregation (2), activates MMP-9 (3), and stimulates increased cytokine production e.g. IL-6 (4). | Stored in azurophil granules (3). | Abundantly released from macrophages, monocytes (5), and epithelial cells (6). | Excluded |
| Proteinase 3 (myeloblastin, PRTN3, C-ANCA antigen, P29, Wegener autoantigen) | Proinflammatory serine protease - facilitates adhesion and transendothelial migration of neutrophils and monocytes (7-9). Potentiates activity of pro-inflammatory mediators e.g. TNF-α and IL-1β, endothelial cell apoptosis (10) and ECM degradation (11). | Constitutively expressed by neutrophils - most abundant in azurophil granules (12, 13). | Small amounts released by monocytes (14), macrophages (15), and endothelial cells (16). | Included |
| Azurocidin (AZU1, CAP37, heparin-binding protein, HBP) | Proinflammatory – induces monocyte and neutrophil proliferation and adhesion via surface β2-integrin (17, 18), phagocytosis, activation of PR3 (19), and secretion of inflammatory cytokines and chemokines e.g. IL-6 and TNF-α (20, 21). Activates macrophage polarisation towards a pro-inflammatory M1 phenotype (22, 23). | Released from azurophil and secretory granules (24, 25). | Secreted in small quantities by epithelial and endothelial cells (18, 26). | Included |
| Myeloperoxidase (MPO) | Facilitates proteolytic degradation of ECM (27), ROS generation (28, 29), and NET formation (30). Contributes to endothelial dysfunction through inhibition of NO (31, 32). Promotes retention of inflammatory neutrophils in circulation via CD11b/CD18 (33). Mediates plaque instability via oxidation of low-density lipoproteins (34, 35). | Primarily from azurophil granules (36, 37) – accounts for 1-5% of total neutrophil protein content (38). | Released from monocytes (39, 40) and macrophages (41), particularly plaque-related macrophages (42). | Included |
| Neutrophil α-defensin 1 (HNP1) | Mediates apoptosis (43), activation and recruitment of neutrophils and monocytes (44-46), increased cytokine and chemokine production e.g. TNF-α and CXCL5 (47). Exacerbates endothelial dysfunction by increased ROS and reduction in NO bioavailability (48). Involved in platelet activation and aggregation (49, 50), leading to plaque instability (51). | Mainly azurophil granules (52, 53) - constitutes up to 7% of total neutrophil protein content (54). | Released from monocytes, natural killer and CD8+ T cells (55). | Included |
| Cathepsin G (CTSG) | Proinflammatory serine protease mediating ECM degradation (56) Acts as a chemoattractant for neutrophils and monocytes via proteolytic modification of CXCL5 and MIP-1δ (CCL15) (57, 58). Involved in platelet activation and inhibits tissue factor plasminogen inhibitor, leading to thrombus formation (59) | Released from azurophil granules (4, 60). | Released by monocytes (61). | Included |
| **Specific (secondary) neutrophil granules** | | | | |
| Neutrophil gelatinase-associated lipocalin (NGAL, lipocalin 2, LCN2, alpha-2-microglobulin-related subunit of MMP-9, oncogene 24p3) | Stimulates increased innate cell proliferation and apoptosis. Modulates ECM degradation by formation of a MMP-9 heterodimer (62) and protects MMP-9 from degradation (63). Markedly enriched in coronary thrombi reflecting proangiogenic lipid binding (64) and plaque destabilising effects (65, 66). | Abundantly released from specific granules of activated neutrophils (67). | Many cells, including endothelial cells and macrophages (67). Specific isoforms released under specific conditions, including from tubular epithelial cells in renal injury (68, 69) and injured cardiomyocytes in failing myocardium (70, 71). | Included |
| Lactoferrin (LTF, lactotransferrin, growth inhibiting protein 12) | Anti-inflammatory – attenuates apoptosis and ROS production (72), inhibits recruitment and adhesion of neutrophils and eosinophils at inflammatory loci (73, 74), increases secretion of anti-inflammatory cytokines e.g. IL-10 and TGF-β (75), and promotes endothelial cell proliferation (76). | Mainly from specific granules of apoptotic neutrophils (77), but also de novo synthesis from migrated neutrophils. | Released by endothelial (78) and epithelial cells [88]. | Included |
| LL-37 (hCAP-18, C-terminal of cathelicidin antimicrobial peptide, CRAMP) | Proinflammatory cathelicidin - stimulates neutrophil proliferation and adhesion (75), secretion of proinflammatory cytokines via IL-1β pathway (79), and NET formation (80). Regulates monocyte extravasation (81, 82) and macrophage polarisation towards M1 phenotype (83). Promotes vascular healing through endothelial cell proliferation (84, 85). | Stored in specific granules (86, 87). | Mainly monocytes (61, 88), macrophages (89), and endothelial cells (16, 90). | Excluded |
| Ficolin-1 (FCN1, ficolin-α, M-ficolin) | Activates the proteolytic complement cascade via the leptin pathway (91). Acts as a chemoattractant for macrophages and neutrophils, stimulates phagocytosis (92), neutrophil adhesion and proliferation (93), and mediates thromboinflammation (94). | Mostly specific granules (95). | Small amounts released by monocytes and macrophages (96) – highly-enriched in the necrotic core of unstable plaques (97). | Included |
| Gelatinase (tertiary) neutrophil granules | | | | |
| Matrix metalloproteinase 8 (MMP-8) | Metalloproteinase involved in the degradation of ECM components, leading to endothelial cell apoptosis (98, 99). Stimulates neutrophil migration via cleavage of CXCR2 ligands e.g. CXCL5 and CXCL8 (100). | Stored in specific and gelatinase granules (3). | Primarily derived from macrophages (3). Many cells including T cells, cardiac fibroblasts (101), and VSMCs (102). | Excluded |
| Matrix metalloproteinase 9 (MMP-9) | Involved in ECM degradation (103, 104), facilitates leukocyte infiltration into the infarct (105), and contributes to plaque destabilisation (106). | Stored in specific and gelatinase granules (103). | Abundantly released from macrophages (107). Also VSMCs (108), fibroblasts (109), and endothelial cells (110). | Excluded |
| Others | | | | |
| Calprotectin (S100A8/A9, MRP-8/14) | Endogenous DAMPs existing as a heterodimeric complex with microbicidal effects (111). Acts as a chemoattractant for neutrophils and other leukocytes (112) and induces migration, proliferation, and activation of innate cells (113, 114). Facilitates generation of ROS, phagocytosis (115), and NETosis (116, 117). Exacerbates production of proinflammatory cytokines (118). Directly mediates myocardial injury by modifying mitochondrial function of myocytes (119, 120). | Markedly enriched in neutrophils (36, 121) - constitutes up to 45% of the total cytosolic protein content (122). | Small amounts released by macrophages and monocytes (123, 124). Also platelets (125), fibroblasts, endothelial cells (126, 127), and VSMCs (128). | Included |
| Neutrophil extracellular traps (NETs) | Composed of proteolytic granule proteins and nuclear components including histones, and double-stranded DNA, which are highly abundant in coronary thrombi (129, 130). Potent microbicidal (131, 132) and prothrombotic capacity through platelet aggregation and fibrin deposition (133). Mediates tissue damage in MI (134). | NETs are only released by neutrophils (135). MPO- and NE-dsDNA components are released mainly by neutrophils. | Release of large amounts dsDNA occurs under specific conditions e.g. necrotic cardiomyocytes and injured tubular epithelial cells. Also mast cells and eosinophils (136, 137). | Included |
| Neutrophil-derived extracellular vesicles (neutrophil-derived microparticles, microvesicles, exosomes, trails) | Small plasma membrane vesicles containing tissue factors e.g. MPO, PR3, NE (138, 139). Both proinflammatory and anti-inflammatory actions – stimulates cytokine release and NET formation (140), cell activation (141), apoptosis and ECM remodelling, partly mediated through IL-1β signalling (142, 143). Regulates endothelial dysfunction (144). | Neutrophil origin characterised by surface expression of neutrophil markers e.g. CD62L, CD63, CD66b, MPO (140, 144). | Many cells, but may lack expression of granulocyte markers - monocytes, endothelial cells and platelets (142, 145). | Included |

Abbreviations: CD, cluster of differentiation; MI, myocardial infarction; NET, neutrophil extracellular traps; ROS, reactive oxygen species; VSMC, vascular smooth muscle cells.

Supplementary Table 2. Search strategy for all databases. Ovid MEDLINE®, Ovid Embase and Embase Classic, Elsevier Scopus, and Clarivate Web of Science databases were searched on 17^th^ September 2021. The Ovid EBM Reviews (Cochrane Central Register of Controlled Trials (CENTRAL)) database was searched on 19^th^ September 2021. The indexed Medical Subject Headings (MeSH) are linked using Boolean operators. String terms are enclosed within parentheses. Asterisked terms (*) signify ‘unlimited wild-card modifier’ functions to capture the range of possible terminological variations. Number of cumulative results are noted in bold, including all preceding searches. Further details of terms used can be found in the Abbreviations section.

| Database | Search details | Coverage |
| --- | --- | --- |
| Ovid MEDLINE® | exp Myocardial Infarction/ 182411  myocardial ischemia/ or acute coronary syndrome/ or coronary occlusion/ or coronary stenosis/ or coronary thrombosis/ 79274  ("myocardial infarction*" or "myocardial ischaemia*" or STEMI or NSTEMI or "N-STEMI" or "Non-STEMI" or "STE-ACS" or "STE-MI" or "ST-elevation-ACS" or "Non-STE-ACS" or "Non-STE-MI" or "Non-ST-elevation-ACS" or "acute coronary syndrome*" or "unstable angina*" or "coronary occlusion*" or "coronary stenos*" or "coronary thrombos*").tw,kf. 237321  1 *or* 2 *or* 3 **342978**  Leukocyte Elastase/ 3938  ("neutrophil elastase*" or "leukocyte elastase*" or HLE or HNE or "polymorphonuclear elastase*" or "PMNelastase*" or "PMN-elastase*" or "PMN-E" or "elastase2" or "Elastase-2" or "granulocyte-derived elastase*").tw,kf. 10942  Peroxidase/ 20815  (MPO or myeloperoxidase or peroxidase).tw,kf. 123858  Lipocalin-2/ 3175  ("neutrophil gelatinase-associated lipocalin*" or "Lipocalin-2" or Lipocalin2 or NGAL or "LCN-2" or LCN2 or HNL or siderocalin*).tw,kf. 5968  Extracellular Traps/ 1945  ("extracellular trap*" or NET or NETS or NETdsDNA or "myeloperoxidase-DNA" or myeloperoxidaseDNA or "MPO-DNA" or "citrullinated histone-3" or CitH3 or "Cit-H3" or "H3Cit-DNA").tw,kf. 133145  exp Leukocyte L1 Antigen Complex/ 4196  (calprotectin* or "S100A8/A9" or S100A8* or "S100-A8*" or "migration inhibitory factor-related protein-8*" or MRP8* or "MRP-8*" or "leukocyte L1 complex*" or "leukocyte L1 subunit*" or "calprotectin L1L subunit*" or "calgranulin-A*" or calgranulinA or "S100 calcium-binding protein A8*").tw,kf. 5641  Myeloblastin/ 1275  (proteinase3* or "proteinase-3*" or PR3* or "PR-3*" or "myeloblastin*" or "azurophil granule protein-7*" or AGP7*).tw,kf. 6981  alpha-Defensins/ 1198  (((neutrophil* or alpha1 or alpha) adj3 defensin*) or (human* adj3 ("neutrophil protein1*" or "neutrophil protein-1*" or "neutrophil peptide*")) or HNP1 or "HNP-1").tw,kf. 1851  (azurocidin* or CAP37* or "CAP-37*" or "cationic protein37*" or "cationic protein-37*" or "heparin-binding protein*" or HBP or "HB-P").tw,kf. 4016  Cathepsin G/ 1100  (cathepsinG* or "cathepsin-G" or cathG* or "cath-G*" or CATG).tw,kf. 2081  Lactoferrin/ 6320  (lactoferrin* or lactotransferrin* or LTF or "growth-inhibiting protein 12*" or lactoferricin* or HLF2 or "HLF-2").tw,kf. 9465  ("ficolin1" or "ficolin-1" or FCN1 or "FCN-1" or "ficolin alpha" or ficolinA or "ficolin-A" or "L-ficolin" or Lficolin or ficolin2 or "ficolin-2" or FCN2 or "FCN-2" or ficolinbeta or "ficolin-beta" or ficolinB or "ficolin-B" or "M-ficolin" or Mficolin).tw,kf. 478  extracellular vesicles/ or cell-derived microparticles/ or exosomes/ 17014  ((neutrophil* or granulocyte* or CD66B* or PMN or polymorphonuclear*) adj3 (microvesicle* or "micro-vesicle*" or microparticle* or "micro-particle*" or "extracellular vesicle*")).tw,kf. 150  *or*/5-26 **320928**  Neutrophils/ 91090  neutrophil*.tw,kf. 155361  28 *or* 29 **181970**  exp Death/ or exp Mortality/ or Secondary prevention/ or Recovery of Function/ or Cardiac Rehabilitation/ or Disease progression/ or Recurrence/ or exp Prognosis/ 2437101  (death* or mortalit* or "fatal outcome*" or MACE or "major adverse cardiovascular event*" or "major adverse cardiac event*" or ACE or "adverse cardiovascular event*" or "adverse cardiac event*" or "long-term outcome*" or recurrence or re-admission or readmission or prognos* or "follow-up" or endpoint*).tw,kf. 3225290  31 *or* 32 **4558178**  4 ***and*** 27 ***and*** 30 ***and*** 33 224  limit 34 to english language 212  limit 35 to "humans only (removes records about animals)" 179 | 1946 to 17th September 2021 |
| Ovid Embase and Embase Classic | exp heart infarction/ 399488  heart muscle ischemia/ or acute coronary syndrome/ or coronary artery obstruction/ or coronary artery occlusion/ or coronary artery thrombosis/ 195088  ("myocardial infarction*" or "myocardial ischaemia*" or STEMI or NSTEMI or "N-STEMI" or "Non-STEMI" or "STE-ACS" or "STE-MI" or "ST-elevation-ACS" or "Non-STE-ACS" or "Non-STE-MI" or "Non-ST-elevation-ACS" or "acute coronary syndrome*" or "unstable angina*" or "coronary occlusion*" or "coronary stenos*" or "coronary thrombos*").tw,kf. 361210  1 *or* 2 *or* 3 **576367**  Leukocyte Elastase/ 7400  ("neutrophil elastase*" or "leukocyte elastase*" or HLE or HNE or "polymorphonuclear elastase*" or "PMNelastase*" or "PMN-elastase*" or "PMN-E" or "elastase2" or "Elastase-2" or "granulocyte-derived elastase*").tw,kf. 14721  Peroxidase/ 32089  (MPO or myeloperoxidase or peroxidase).tw,kf. 143350  neutrophil gelatinase associated lipocalin/ 10396  ("neutrophil gelatinase-associated lipocalin*" or "Lipocalin-2" or Lipocalin2 or NGAL or "LCN-2" or LCN2 or HNL or siderocalin*).tw,kf. 10113  Extracellular Trap/ 5465  ("extracellular trap*" or NET or NETS or NETdsDNA or "myeloperoxidase-DNA" or myeloperoxidaseDNA or "MPO-DNA" or "citrullinated histone-3" or CitH3 or "Cit-H3" or "H3Cit-DNA").tw,kf. 163583  calgranulin/ 10279  (calprotectin* or "S100A8/A9" or S100A8* or "S100-A8*" or "migration inhibitory factor-related protein-8*" or MRP8* or "MRP-8*" or "leukocyte L1 complex*" or "leukocyte L1 subunit*" or "calprotectin L1L subunit*" or "calgranulin-A*" or calgranulinA or "S100 calcium-binding protein A8*").tw,kf. 11810  Myeloblastin/ 3394  (proteinase3* or "proteinase-3*" or PR3* or "PR-3*" or "myeloblastin*" or "azurophil granule protein-7*" or AGP7*).tw,kf. 11312  alpha defensin/ 2091  (((neutrophil* or alpha1 or alpha) adj3 defensin*) or (human* adj3 ("neutrophil protein1*" or "neutrophil protein-1*" or "neutrophil peptide*")) or HNP1 or "HNP-1").tw,kf. 2313  azurocidin/ 286  (azurocidin* or CAP37* or "CAP-37*" or "cationic protein37*" or "cationic protein-37*" or "heparin-binding protein*" or HBP or "HB-P").tw,kf. 6018  Cathepsin G/ 2406  (cathepsinG* or "cathepsin-G" or cathG* or "cath-G*" or CATG).tw,kf. 2510  Lactoferrin/ 11315  (lactoferrin* or lactotransferrin* or LTF or "growth-inhibiting protein 12*" or lactoferricin* or HLF2 or "HLF-2").tw,kf. 11299  ("ficolin1" or "ficolin-1" or FCN1 or "FCN-1" or "ficolin alpha" or ficolinA or "ficolin-A" or "L-ficolin" or Lficolin or ficolin2 or "ficolin-2" or FCN2 or "FCN-2" or ficolinbeta or "ficolin-beta" or ficolinB or "ficolin-B" or "M-ficolin" or Mficolin).tw,kf. 731  exosome/ or membrane microparticle/ 41457  ((neutrophil* or granulocyte* or CD66B* or PMN or polymorphonuclear*) adj3 (microvesicle* or "micro-vesicle*" or microparticle* or "micro-particle*" or "extracellular vesicle*")).tw,kf. 259  *or*/5-26 **428885**  neutrophil/ 150195  neutrophil*.tw,kf. 224327  29 *or* 30 **259714**  exp death/ or mortality/ or fatal outcome/ or disease progression/ or recurrence/ or exp prognosis/ 2421016  (death* or mortalit* or "fatal outcome*" or MACE or "major adverse cardiovascular event*" or "major adverse cardiac event*" or "long-term outcome*" or recurrence or re-admission or readmission or prognos*).tw,kf. 3531837  32 *or* 33 **4279621**  4 ***and*** 28 ***and*** 31 ***and*** 34 401  limit 35 to english language 387  limit 36 to "humans only (removes records about animals)" 323  limit 37 to conference abstracts 127  37 not 38 196 | 1947 to 17th September 2021 |
| Cochrane CENTRAL | exp Myocardial Infarction/ 11056  myocardial ischemia/ or acute coronary syndrome/ or coronary occlusion/ or coronary stenosis/ or coronary thrombosis/ 7521  ("myocardial infarction*" or "myocardial ischaemia*" or STEMI or NSTEMI or "N-STEMI" or "Non-STEMI" or "STE-ACS" or "STE-MI" or "ST-elevation-ACS" or "Non-STE-ACS" or "Non-STE-MI" or "Non-ST-elevation-ACS" or "acute coronary syndrome*" or "unstable angina*" or "coronary occlusion*" or "coronary stenos*" or "coronary thrombos*").mp. 40407  1 *or* 2 *or* 3 **43358**  Leukocyte Elastase/ 182  ("neutrophil elastase*" or "leukocyte elastase*" or HLE or HNE or "polymorphonuclear elastase*" or "PMNelastase*" or "PMN-elastase*" or "PMN-E" or "elastase2" or "Elastase-2" or "granulocyte-derived elastase*").mp. 629  Peroxidase/ 253  (MPO or myeloperoxidase or peroxidase).mp. 3175  Lipocalin-2/ 117  ("neutrophil gelatinase-associated lipocalin*" or "Lipocalin-2" or Lipocalin2 or NGAL or "LCN-2" or LCN2 or HNL or siderocalin*).mp. 999  Extracellular Traps/ 7  ("extracellular trap*" or NET or NETS or NETdsDNA or "myeloperoxidase-DNA" or myeloperoxidaseDNA or "MPO-DNA" or "citrullinated histone-3" or CitH3 or "Cit-H3" or "H3Cit-DNA").mp. 9939  exp Leukocyte L1 Antigen Complex/ 152  (calprotectin* or "S100A8/A9" or S100A8* or "S100-A8*" or CP or "migration inhibitory factor-related protein-8*" or MRP8* or "MRP-8*" or "leukocyte L1 complex*" or "leukocyte L1 subunit*" or "calprotectin L1L subunit*" or "calgranulin-A*" or calgranulinA or "S100 calcium-binding protein A8*").mp. 7057  Myeloblastin/ 17  (proteinase3* or "proteinase-3*" or PR3* or "PR-3*" or "myeloblastin*" or "azurophil granule protein-7*" or AGP7*).mp. 681  alpha-Defensins/ 11  (((neutrophil* or alpha1 or alpha) adj3 defensin*) or (human* adj3 ("neutrophil protein1*" or "neutrophil protein-1*" or "neutrophil peptide*")) or HNP1 or "HNP-1").mp. 63  (azurocidin* or CAP37* or "CAP-37*" or "cationic protein37*" or "cationic protein-37*" or "heparin-binding protein*" or HBP or "HB-P").mp. 370  Cathepsin G/ 5  (cathepsinG* or "cathepsin-G" or cathG* or "cath-G*" or CATG).mp. 41  Lactoferrin/ 277  (lactoferrin* or lactotransferrin* or LTF or "growth-inhibiting protein 12*" or lactoferricin* or HLF2 or "HLF-2").mp. 771  ("ficolin1" or "ficolin-1" or FCN1 or "FCN-1" or "ficolin alpha" or ficolinA or "ficolin-A" or "L-ficolin" or Lficolin or ficolin2 or "ficolin-2" or FCN2 or "FCN-2" or ficolinbeta or "ficolin-beta" or ficolinB or "ficolin-B" or "M-ficolin" or Mficolin).mp. 32  extracellular vesicles/ or cell-derived microparticles/ or exosomes/ 110  ((neutrophil* or granulocyte* or CD66B* or PMN or polymorphonuclear*) adj3 (microvesicle* or "micro-vesicle*" or microparticle* or "micro-particle*" or "extracellular vesicle*")).mp. 6  *or*/5-26 **23276**  Neutrophils/ 1423  neutrophil*.mp. 12979  28 *or* 29 **12979**  exp death/ or mortality/ or fatal outcome/ or disease progression/ or recurrence/ or exp prognosis/ 174189  (death* or mortalit* or "fatal outcome*" or MACE or "major adverse cardiovascular event*" or "major adverse cardiac event*" or "long-term outcome*" or recurrence or "re-admission" or readmission or prognos*).mp. 232611  31 *or* 32 **354817**  4 ***and*** 27 ***and*** 30 88  33 ***and*** 34 44  limit 35 to English language 31 | 1998 to 19th September 2021 |
| Elsevier Scopus | ( ( ( TITLE-ABS-KEY ( "myocardial infarction*" OR "myocardial ischaemia*" OR stemi OR nstemi OR "N-STEMI" OR "Non-STEMI" OR "STE-ACS" OR "STE-MI" OR "ST-elevation-ACS" OR "Non-STE-ACS" OR "Non-STE-MI" OR "Non-ST-elevation-ACS" OR "acute coronary syndrome*" OR "unstable angina*" OR "coronary occlusion*" OR "coronary stenos*" OR "coronary thrombos*" ) ***AND*** TITLE-ABS-KEY ( "neutrophil elastase*" OR "leukocyte elastase*" OR hle OR hne OR "polymorphonuclear elastase*" OR "PMNelastase*" OR "PMN-elastase*" OR "PMN-E" OR "elastase2" OR "Elastase-2" OR "granulocyte-derived elastase*" OR mpo OR myeloperoxidase OR peroxidase OR "neutrophil gelatinase-associated lipocalin*" OR "Lipocalin-2" OR lipocalin2 OR ngal OR "LCN-2" OR lcn2 OR hnl OR siderocalin* OR "extracellular trap*" OR net OR nets OR netdsdna OR "myeloperoxidase-DNA" OR myeloperoxidasedna OR "MPO-DNA" OR "citrullinated histone-3" OR cith3 OR "Cit-H3" OR "H3Cit-DNA" OR calprotectin* OR "S100A8/A9" OR s100a8* OR "S100-A8*" OR "migration inhibitory factor-related protein-8*" OR mrp8* OR "MRP-8*" OR "leukocyte L1 complex*" OR "leukocyte L1 subunit*" OR "calprotectin L1L subunit*" OR "calgranulin-A*" OR calgranulina OR "S100 calcium-binding protein A8*" OR proteinase3* OR "proteinase-3*" OR pr3* OR "PR-3*" OR "myeloblastin*" OR "azurophil granule protein-7*" OR agp7* OR ( ( neutrophil* OR alpha1 OR alpha ) W/3 defensin* ) OR ( human* W/3 ( "neutrophil protein1*" OR "neutrophil protein-1*" OR "neutrophil peptide*" ) ) OR hnp1 OR "HNP-1" OR azurocidin* OR cap37* OR "CAP-37*" OR "cationic protein37*" OR "cationic protein-37*" OR "heparin-binding protein*" OR hbp OR "HB-P" OR cathepsing* OR "cathepsin-G" OR cathg* OR "cath-G*" OR catg OR lactoferrin* OR lactotransferrin* OR ltf OR "growth-inhibiting protein 12*" OR lactoferricin* OR hlf2 OR "HLF-2" OR "ficolin1" OR "ficolin-1" OR fcn1 OR "FCN-1" OR "ficolin alpha" OR ficolina OR "ficolin-A" OR "L-ficolin" OR lficolin OR ficolin2 OR "ficolin-2" OR fcn2 OR "FCN-2" OR ficolinbeta OR "ficolin-beta" OR ficolinb OR "ficolin-B" OR "M-ficolin" OR mficolin OR ( ( neutrophil* OR granulocyte* OR cd66b* OR pmn OR polymorphonuclear* ) W/3 ( microvesicle* OR "micro-vesicle*" OR microparticle* OR "micro-particle*" OR "extracellular vesicle*" ) ) ) ***AND*** TITLE-ABS-KEY ( neutrophil* ) ) ) ***AND*** ( TITLE-ABS-KEY ( death* OR mortalit* OR "fatal outcome*" OR mace OR "major adverse cardiovascular event*" OR "major adverse cardiac event*" OR "long-term outcome*" OR recurrence OR re-admission OR readmission OR prognos* ) ) ) ***AND NOT*** ( ( ( TITLE ( mice OR murine OR mouse OR rat OR rats OR "Sprague Dawley" ) OR ABS ( mice OR murine OR mouse OR rat OR rats OR "Sprague Dawley" ) ) ) ) ***AND*** ( LIMIT-TO ( LANGUAGE , "English" ) ) ***AND*** ( EXCLUDE ( EXACTKEYWORD , "Nonhuman" ) OR EXCLUDE ( EXACTKEYWORD , "Animals" ) OR EXCLUDE ( EXACTKEYWORD , "Animal Experiment" ) OR EXCLUDE ( EXACTKEYWORD , "Animal Model" ) OR EXCLUDE ( EXACTKEYWORD , "Animal" ) OR EXCLUDE ( EXACTKEYWORD , "Animal Tissue" ) OR EXCLUDE ( EXACTKEYWORD , "Rat" ) OR EXCLUDE ( EXACTKEYWORD , "Mouse" ) OR EXCLUDE ( EXACTKEYWORD , "Animal Cell" ) OR EXCLUDE ( EXACTKEYWORD , "Rats" ) OR EXCLUDE ( EXACTKEYWORD , "Mice" ) ) **199** | 1966 to 17th September 2021 |
| Clarivate Web of Science | (((((TS=("myocardial infarction*" OR "myocardial ischaemia*" OR stemi OR nstemi OR "N-STEMI" OR "Non-STEMI" OR "STE-ACS" OR "STE-MI" OR "ST-elevation-ACS" OR "Non-STE-ACS" OR "Non-STE-MI" OR "Non-ST-elevation-ACS" OR "acute coronary syndrome*" OR "unstable angina*" OR "coronary occlusion*" OR "coronary stenos*" OR "coronary thrombos*")) ***AND*** TS=( "neutrophil elastase*" OR "leukocyte elastase*" OR hle OR hne OR "polymorphonuclear elastase*" OR "PMNelastase*" OR "PMN-elastase*" OR "PMN-E" OR "elastase2" OR "Elastase-2" OR "granulocyte-derived elastase*" OR mpo OR myeloperoxidase OR peroxidase OR "neutrophil gelatinase-associated lipocalin*" OR "Lipocalin-2" OR lipocalin2 OR ngal OR "LCN-2" OR lcn2 OR hnl OR siderocalin* OR "extracellular trap*" OR net OR nets OR netdsdna OR "myeloperoxidase-DNA" OR myeloperoxidasedna OR "MPO-DNA" OR "citrullinated histone-3" OR cith3 OR "Cit-H3" OR "H3Cit-DNA" OR calprotectin* OR "S100A8/A9" OR s100a8* OR "S100-A8*" OR "migration inhibitory factor-related protein-8*" OR mrp8* OR "MRP-8*" OR "leukocyte L1 complex*" OR "leukocyte L1 subunit*" OR "calprotectin L1L subunit*" OR "calgranulin-A*" OR calgranulina OR "S100 calcium-binding protein A8*" OR proteinase3* OR "proteinase-3*" OR pr3* OR "PR-3*" OR "myeloblastin*" OR "azurophil granule protein-7*" OR agp7* OR ( ( neutrophil* OR alpha1 OR alpha ) NEAR/3 defensin* ) OR ( human* NEAR/3 ( "neutrophil protein1*" OR "neutrophil protein-1*" OR "neutrophil peptide*" ) ) OR hnp1 OR "HNP-1" OR azurocidin* OR cap37* OR "CAP-37*" OR "cationic protein37*" OR "cationic protein-37*" OR "heparin-binding protein*" OR hbp OR "HB-P" OR cathepsing* OR "cathepsin-G" OR cathg* OR "cath-G*" OR catg OR lactoferrin* OR lactotransferrin* OR ltf OR "growth-inhibiting protein 12*" OR lactoferricin* OR hlf2 OR "HLF-2" OR "ficolin1" OR "ficolin-1" OR fcn1 OR "FCN-1" OR "ficolin alpha" OR ficolina OR "ficolin-A" OR "L-ficolin" OR lficolin OR ficolin2 OR "ficolin-2" OR fcn2 OR "FCN-2" OR ficolinbeta OR "ficolin-beta" OR ficolinb OR "ficolin-B" OR "M-ficolin" OR mficolin OR ( ( neutrophil* OR granulocyte* OR cd66b* OR pmn OR polymorphonuclear* ) NEAR/3 ( microvesicle* OR "micro-vesicle*" OR microparticle* OR "micro-particle*" OR "extracellular vesicle*")))) ***AND*** TS=(neutrophil*))) ***AND*** TS=(death* OR mortalit* OR "fatal outcome*" OR MACE OR "major adverse cardiovascular event*" OR "major adverse cardiac event*" OR "long-term outcome*" OR recurrence OR re-admission OR readmission OR prognos*)) ***NOT***  TS=(mice OR murine OR mouse OR rat OR rats OR "Sprague Dawley") **229** | 1900 to 17th September 2021 |

Supplementary Table 3. Supplementary search strategy.

| Author (Year) | From reference list (backward tracking) | Citing study, as of 30th September 2021 (forward tracking) | Study included, or reason for exclusion |
| --- | --- | --- | --- |
| Baldus et al. (2003)(146) | Brügger-Andersen et al. (2008)(147) |  | Included |
|  | Cavusoglu et al. (2007)(148) |  | Included |
|  | McCann et al. (2009)(149) |  | Included |
|  | Mocatta et al. (2007)(150) |  | Included |
|  | Nicholls et al. (2011)(151) |  | Population criteria not appropriate |
|  | Scirica et al. (2010)(152) |  | Included |
| Brennan et al. (2003)(153) | Apple et al. (2011)(154) |  | Population criteria not appropriate |
|  | Kaya et al. (2012)(155) |  | Included |
|  | Roman et al. (2010)(156) |  | Inadequate clinical endpoint |
|  | Scharnagl et al. (2014)(157) |  | Population criteria not appropriate |
| Yndestad et al. (2009)(70) | Giurgea et al. (2020)(158) |  | Population criteria not appropriate |
| Helanova et al. (2015)(159) |  | Yndestad et al. (2009)(70) | Included |
| Langseth et al. (2020)(160) |  | Helseth et al. (2019)(161) | Included |
|  |  | Morrow et al. (2008)(162) | Included |
|  |  | Rainer et al. (2006)(163) | No measurement of neutrophil-derived prognostic factor |
| Nymo et al. (2018)(164) |  | Katagiri et al. (2016)(165) | Population criteria not appropriate and Inadequate clinical endpoint |

Supplementary Table 4. Quality assessment for included cohort studies.

| Study | Defined selection criteria | Attrition rate | Defined outcome | Blinded measurement and outcome assessment | Objective outcome reporting | Completeness of data | Appropriate cut point^‡^ | Adjusted for confounders | Overall quality^†^ |
| --- | --- | --- | --- | --- | --- | --- | --- | --- | --- |
| Liu et al. (2021)(166) | Y | 2.4% | Y | Y | Y | 100% | Y | Y | + |
| Avci et al. (2020)(167) | Y | 0% | Y | Y | Y | 100% | Y | Y | + |
| Obeid et al. (2020)(168) | Y | 1.7% | Y | Y* | Y | 100% | Y | Y | + |
| Langseth et al. (2020)(160) | Y | 0% | Y | Y | Y | 99.7% | N | Y | + |
| Peng et al. (2019)(169) | Y | 2.8% | Y | U | U | 100% | Y | Y | + |
| Nguyen et al. (2019)(170) | Y | NR | Y | U | U | 100% | N | Y | 0 |
| Wang et al. (2019)(171) | Y | 0% | Y | U | U | 100% | N | Y | + |
| Helseth et al. (2019)(161) | Y | 7.7% | Y | Y | Y | 92.3% | N | Y | + |
| Nymo et al. (2019)(164) | Y | 0% | Y | Y | Y | 100% | N | Y | + |
| Wang et al. (2018)(172) | Y | 0% | Y | Y | Y | 100% | N | Y | + |
| Barbarash et al. (2017)(173) | Y | 10.9% | Y | U | Y | 100% | U | Y | + |
| Helanova et al. (2015)(159) | Y | 0% | Y | Y | Y | 100% | Y | Y | + |
| Akcay et al. (2012)(174) | Y | 0% | Y | U | Y | 100% | Y | Y | + |
| Alfakry et al. (2012)(175) | Y | 0% | Y | Y | Y | 100% | N | U | 0 |
| Lindberg et al. (2012)(176) | Y | 0% | Y | Y | Y | 100% | N | Y | + |
| Ng et al. (2011)(177) | Y | 0% | Y | Y | Y | NR | N | Y | + |
| Scirica et al. (2010)(152) | Y | 0% | Y | Y* | Y | 100% | U | Y | + |
| Jensen et al. (2010)(178) | Y | 0% | Y | Y | U | 100% | Y | Y | + |
| Yndestad et al. (2009)(70) | Y | 0% | Y | Y* | Y | 100% | N | U | 0 |
| McCann et al. (2009)(149) | Y | 1% | Y | U | N | 92% | N | Y | + |
| Morrow et al. (2008) | Y | 0% | Y | Y* | Y | 100% | N | Y | + |
| Brügger-Andersen et al. (2008)(147) | Y | 0% | Y | Y | Y | 99.8% | N | Y | + |
| Cavusoglu et al. (2007)(148) | Y | 0% | Y | Y | Y | 94.3% | N | Y | + |
| Baldus et al. (2003)(146) | Y | 0% | Y | Y* | Y | 100% | N | Y | + |

Key: Y = Yes; N = no; NR = not reported; U = unclear. *Denotes use of an independent endpoint committee; †Overall quality, as assessed by risk of bias and completeness of adjustments made for potential confounders; ++ = high quality; + = acceptable quality; 0 = poor quality; ‡Study summary statistics reported using continuous data or grouped according to appropriate and valid cut-points, i.e., informed by ROC curve analysis.

Supplementary Table 5. Quality assessment of case-control studies.

| Study (Year) | Defined selection criteria | Defined outcomes | Blinded measurement and outcome assessment | Objective outcome reporting | Appropriate cut point^‡^ | Adjusted for confounders | Overall quality^†^ |
| --- | --- | --- | --- | --- | --- | --- | --- |
| Hally et al. (2021)(179) | Y | Y | NA | Y | Y | Y | + |
| Kaya et al. (2012)(155) | Y | Y | U | Y | Y | Y | + |
| Mocatta et al. (2007)(150) | Y | Y | Y | Y | Y | Y | + |

Key: Y = Yes, N = No, NA = Not appliable, U = Unclear. †Overall quality assessed by risk of bias and completeness of adjustments made to potential confounders; ++ = high quality, + = acceptable quality, 0 = poor quality; ‡Study summary statistics reported using continuous data or grouped according to appropriate and valid cut-points, i.e., informed by ROC curve analysis.

Supplementary Figure 1. Eligibility screen tool. The individual components included in the tool were developed from the inclusion criteria as follows: the study population comprised ≥70% patients with confirmed ACS; neutrophil-enriched biomarkers were sampled during hospital admission with ACS; and outcomes included, at minimum, all-cause mortality at ≥6 months following admission.

Supplementary figure 2. Quality assessment tool for cohort studies. This form, adapted from the SIGN, NOS, and QUIPS tools for observational cohort studies, was used to assess the internal validity and risk of bias of the cohort studies included in the review.


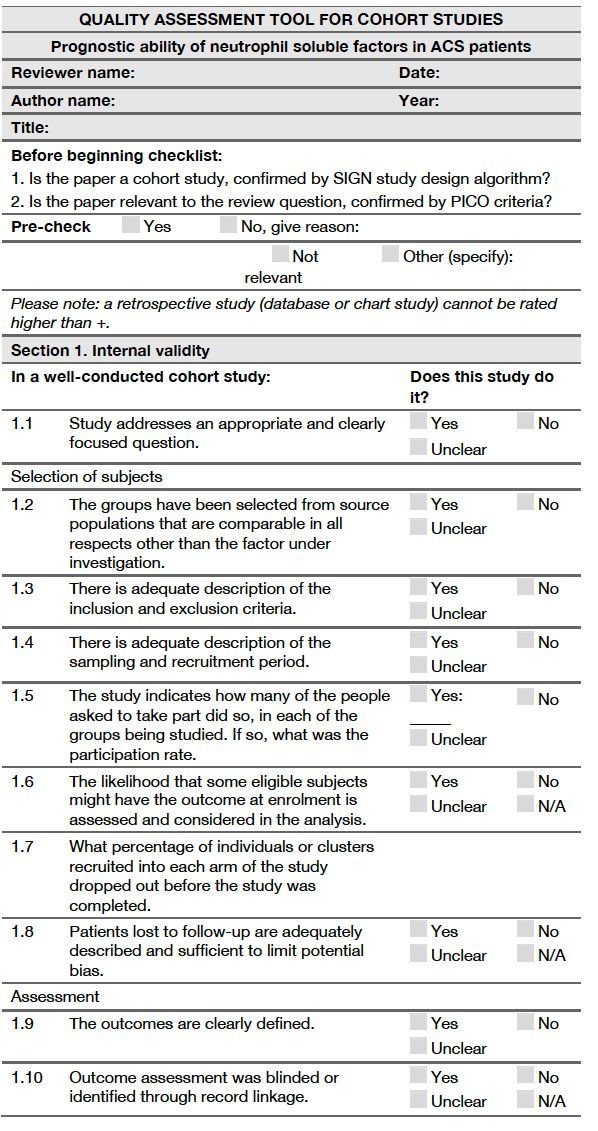

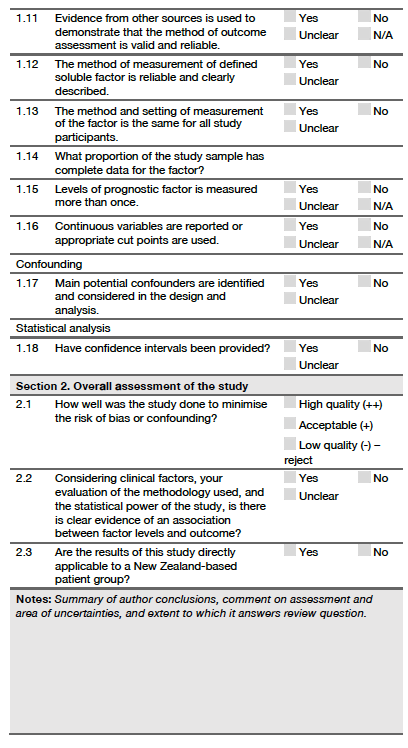


Supplementary figure 3. Quality assessment tool for case-control studies. This form, adapted from the SIGN, NOS, QUIPS, and JBI tools for case-control studies, was used to assess the internal validity and risk of bias of the case-control studies included in the present review.

Supplementry Figure 4. Standard funnel plots of A) MPO and B) NGAL studies, with the risk ratio (x axis) plotted against the standard error of the risk ratio (y axis).

1. MPO

B) NGAL

**
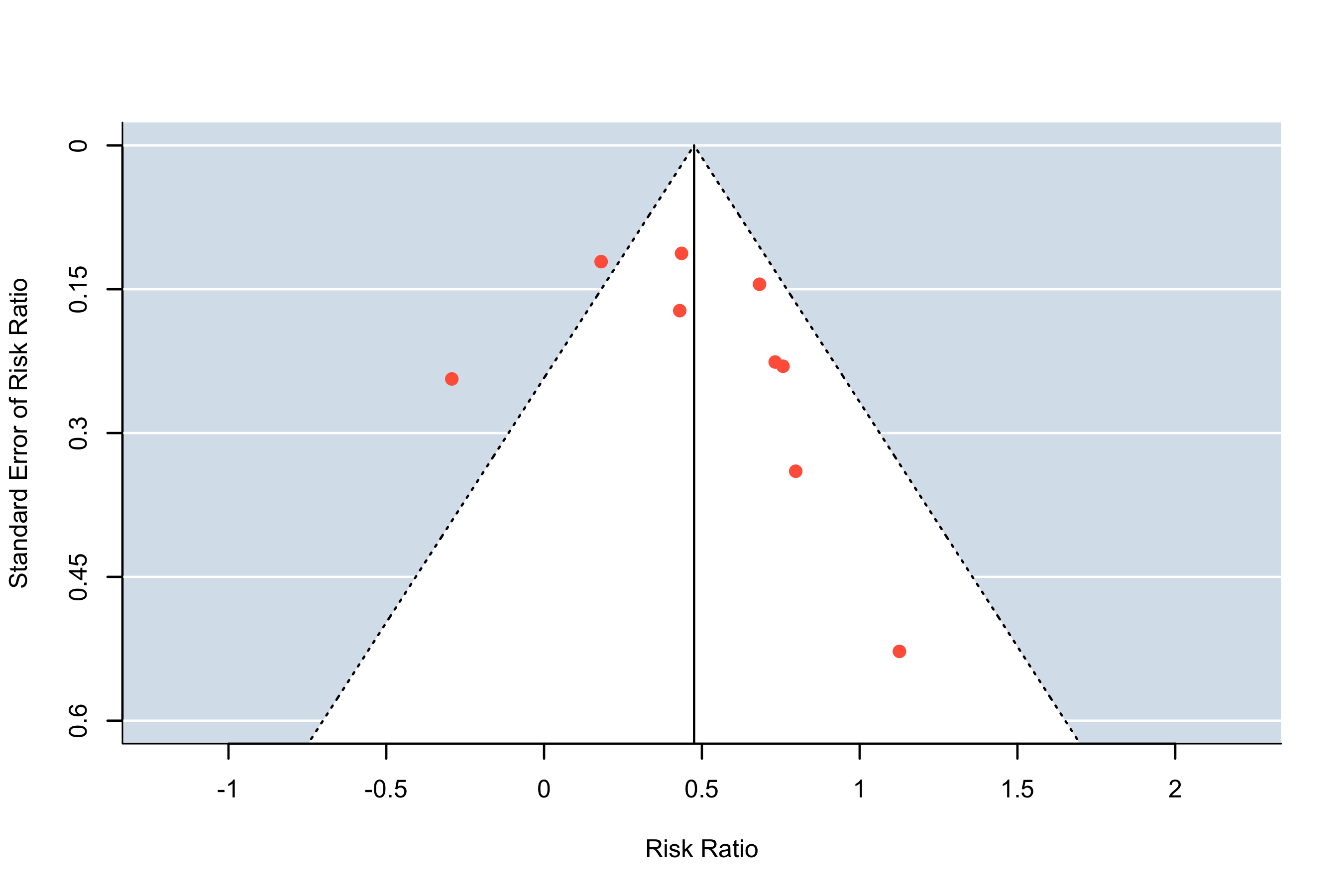
**

**
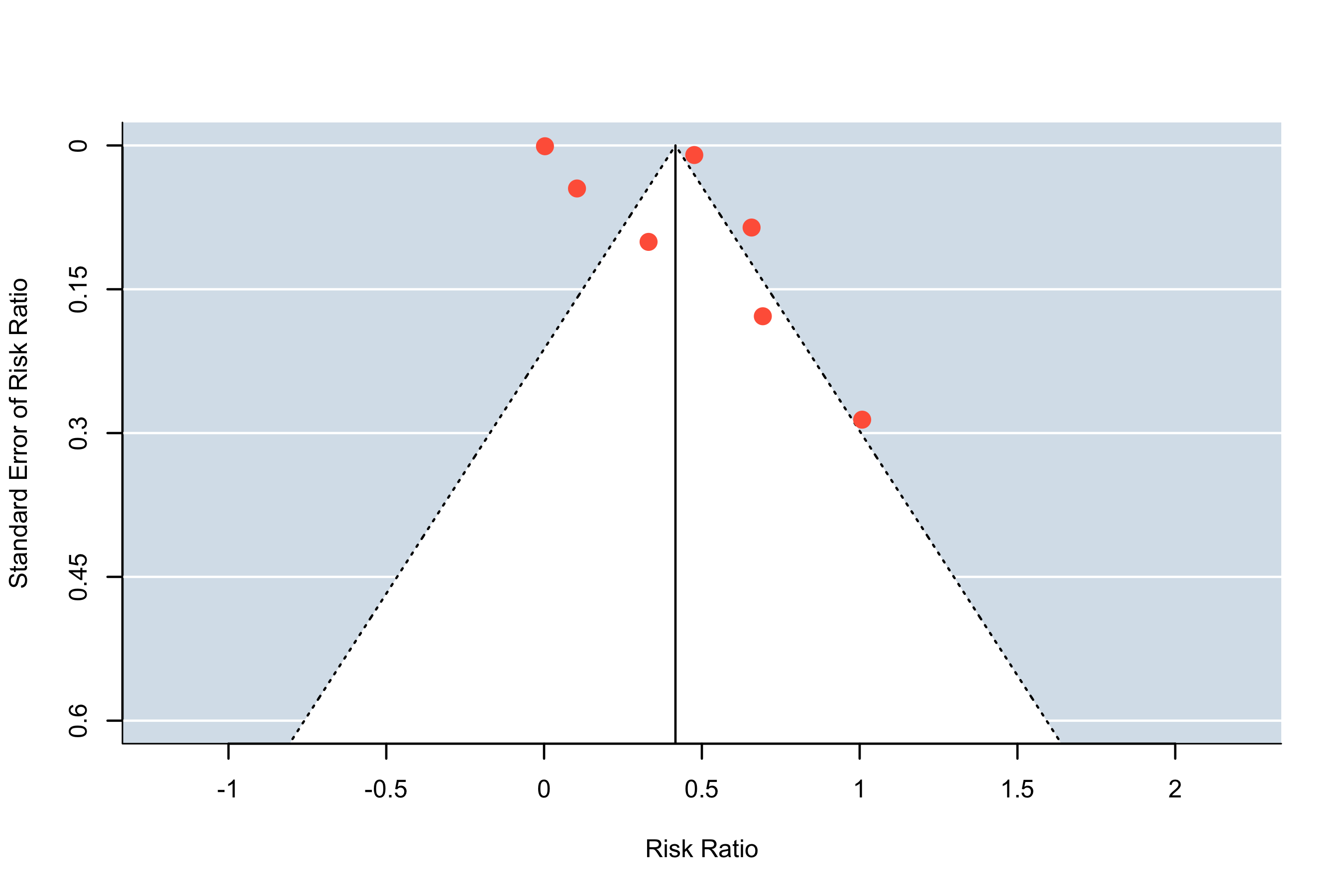
**

**References.**

1. Papayannopoulos V, Metzler KD, Hakkim A, Zychlinsky A. Neutrophil elastase and myeloperoxidase regulate the formation of neutrophil extracellular traps. Journal of Cell Biology. 2010;191(3):677-91.

2. Massberg S, Grahl L, von Bruehl ML, Manukyan D, Pfeiler S, Goosmann C, et al. Reciprocal coupling of coagulation and innate immunity via neutrophil serine proteases. Nat Med. 2010;16(8):887-96.

3. Leclercq A, Houard X, Philippe M, Ollivier V, Sebbag U, Meilhac O, et al. Involvement of intraplaque hemorrhage in atherothrombosis evolution via neutrophil protease enrichment. Journal of Leukocyte Biology. 2007;82(6):1420-9.

4. Pham CTN. Neutrophil serine proteases: specific regulators of inflammation. Nature Reviews Immunology. 2006;6(7):541-50.

5. Dollery CM, Owen CA, Sukhova GK, Krettek A, Shapiro SD, Libby P. Neutrophil elastase in human atherosclerotic plaques: production by macrophages. Circulation. 2003;107(22):2829-36.

6. Houghton AM, Rzymkiewicz DM, Ji H, Gregory AD, Egea EE, Metz HE, et al. Neutrophil elastase-mediated degradation of IRS-1 accelerates lung tumor growth. Nat Med. 2010;16(2):219-23.

7. Brachemi S, Mambole A, Fakhouri F, Mouthon L, Guillevin L, Lesavre P, et al. Increased membrane expression of proteinase 3 during neutrophil adhesion in the presence of anti proteinase 3 antibodies. J Am Soc Nephrol. 2007;18(8):2330-9.

8. Kuckleburg CJ, Tilkens SB, Santoso S, Newman PJ. Proteinase 3 contributes to transendothelial migration of NB1-positive neutrophils. J Immunol. 2012;188(5):2419-26.

9. Taekema-Roelvink MEJ, Kooten CV, Kooij SV, Heemskerk E, Daha MR. Proteinase 3 enhances endothelial monocyte chemoattractant protein-1 production and induces increased adhesion of neutrophils to endothelial cells by upregulating intercellular cell adhesion molecule-1. J Am Soc Nephrol. 2001;12(5):932-40.

10. Yang JJ, Preston GA, Pendergraft WF, Segelmark M, Heeringa P, Hogan SL, et al. Internalization of Proteinase 3 Is Concomitant with Endothelial Cell Apoptosis and Internalization of Myeloperoxidase with Generation of Intracellular Oxidants. The American Journal of Pathology. 2001;158(2):581-92.

11. Rao NV, Wehner NG, Marshall BC, Gray WR, Gray BH, Hoidal JR. Characterization of proteinase-3 (PR-3), a neutrophil serine proteinase. Structural and functional properties. J Biol Chem. 1991;266(15):9540-8.

12. Witko-Sarsat V, Cramer EM, Hieblot C, Guichard J, Nusbaum P, Lopez S, et al. Presence of proteinase 3 in secretory vesicles: evidence of a novel, highly mobilizable intracellular pool distinct from azurophil granules. Blood. 1999;94(7):2487-96.

13. Csernok E, Ernst M, Schmitt W, Bainton DF, Gross WL. Activated neutrophils express proteinase 3 on their plasma membrane in vitro and in vivo. Clin Exp Immunol. 1994;95(2):244-50.

14. van der Geld YM, Limburg PC, Kallenberg CG. Proteinase 3, Wegener's autoantigen: from gene to antigen. J Leukoc Biol. 2001;69(2):177-90.

15. Kantari C, Pederzoli-Ribeil M, Amir-Moazami O, Gausson-Dorey V, Moura IC, Lecomte MC, et al. Proteinase 3, the Wegener autoantigen, is externalized during neutrophil apoptosis: evidence for a functional association with phospholipid scramblase 1 and interference with macrophage phagocytosis. Blood. 2007;110(12):4086-95.

16. Mayet WJ, Csernok E, Szymkowiak C, Gross WL, Meyer zum Büschenfelde KH. Human endothelial cells express proteinase 3, the target antigen of anticytoplasmic antibodies in Wegener's granulomatosis. Blood. 1993;82(4):1221-9.

17. Soehnlein O, Zernecke A, Eriksson EE, Rothfuchs AG, Pham CT, Herwald H, et al. Neutrophil secretion products pave the way for inflammatory monocytes. Blood. 2008;112(4):1461-71.

18. Lee TD, Gonzalez ML, Kumar P, Grammas P, Pereira HA. CAP37, a neutrophil-derived inflammatory mediator, augments leukocyte adhesion to endothelial monolayers. Microvasc Res. 2003;66(1):38-48.

19. Zhang J, Alcaide P, Liu L, Sun J, He A, Luscinskas FW, et al. Regulation of Endothelial Cell Adhesion Molecule Expression by Mast Cells, Macrophages, and Neutrophils. PLoS One. 2011;6(1):e14525.

20. Heinzelmann M, Platz A, Flodgaard H, Polk HC, Jr., Miller FN. Endocytosis of heparin-binding protein (CAP37) is essential for the enhancement of lipopolysaccharide-induced TNF-alpha production in human monocytes. J Immunol. 1999;162(7):4240-5.

21. Heinzelmann M, Kim E, Hofmeister A, Gordon LE, Platz A, Cheadle WG. Heparin binding protein (CAP37) differentially modulates endotoxin-induced cytokine production. Int J Surg Investig. 2001;2(6):457-66.

22. Rasmussen PB, Bjørn S, Hastrup S, Nielsen PF, Norris K, Thim L, et al. Characterization of recombinant human HBP/CAP37/azurocidin, a pleiotropic mediator of inflammation-enhancing LPS-induced cytokine release from monocytes. FEBS Lett. 1996;390(1):109-12.

23. Soehnlein O, Lindbom L. Neutrophil-derived azurocidin alarms the immune system. J Leukoc Biol. 2009;85(3):344-51.

24. Tapper H, Karlsson A, Mörgelin M, Flodgaard H, Herwald H. Secretion of heparin-binding protein from human neutrophils is determined by its localization in azurophilic granules and secretory vesicles. Blood. 2002;99(5):1785-93.

25. Soehnlein O, Lindbom L. Neutrophil-derived azurocidin alarms the immune system. Journal of Leukocyte Biology. 2009;85(3):344-51.

26. Lee TD, Gonzalez ML, Kumar P, Chary-Reddy S, Grammas P, Pereira HA. CAP37, a novel inflammatory mediator: its expression in endothelial cells and localization to atherosclerotic lesions. Am J Pathol. 2002;160(3):841-8.

27. Ali M, Pulli B, Courties G, Tricot B, Sebas M, Iwamoto Y, et al. Myeloperoxidase Inhibition Improves Ventricular Function and Remodeling After Experimental Myocardial Infarction. JACC Basic Transl Sci. 2016;1(7):633-43.

28. Klebanoff SJ, Kettle AJ, Rosen H, Winterbourn CC, Nauseef WM. Myeloperoxidase: a front-line defender against phagocytosed microorganisms. Journal of Leukocyte Biology. 2013;93(2):185-98.

29. Zhang R, Brennan ML, Fu X, Aviles RJ, Pearce GL, Penn MS, et al. Association between myeloperoxidase levels and risk of coronary artery disease. Jama. 2001;286(17):2136-42.

30. Parker H, Albrett AM, Kettle AJ, Winterbourn CC. Myeloperoxidase associated with neutrophil extracellular traps is active and mediates bacterial killing in the presence of hydrogen peroxide. J Leukoc Biol. 2012;91(3):369-76.

31. Eiserich JP, Baldus S, Brennan M-L, Ma W, Zhang C, Tousson A, et al. Myeloperoxidase, a leukocyte-derived vascular NO oxidase. Science. 2002;296(5577):2391-4.

32. Baldus S, Heitzer T, Eiserich JP, Lau D, Mollnau H, Ortak M, et al. Myeloperoxidase enhances nitric oxide catabolism during myocardial ischemia and reperfusion. Free Radic Biol Med. 2004;37(6):902-11.

33. El Kebir D, József L, Pan W, Filep JnG. Myeloperoxidase delays neutrophil apoptosis through CD11b/CD18 integrins and prolongs inflammation. Circulation research. 2008;103(4):352-9.

34. Naruko T, Furukawa A, Yunoki K, Komatsu R, Nakagawa M, Matsumura Y, et al. Increased expression and plasma levels of myeloperoxidase are closely related to the presence of angiographically-detected complex lesion morphology in unstable angina. Heart. 2010;96(21):1716-22.

35. Podrez EA, Schmitt D, Hoff HF, Hazen SL. Myeloperoxidase-generated reactive nitrogen species convert LDL into an atherogenic form in vitro. The Journal of Clinical Investigation. 1999;103(11):1547-60.

36. Miyamoto S, Ueda M, Ikemoto M, Naruko T, Itoh A, Tamaki S, et al. Increased serum levels and expression of S100A8/A9 complex in infiltrated neutrophils in atherosclerotic plaque of unstable angina. Heart. 2008;94(8):1002-7.

37. Sugiyama S, Okada Y, Sukhova GK, Virmani R, Heinecke JW, Libby P. Macrophage myeloperoxidase regulation by granulocyte macrophage colony-stimulating factor in human atherosclerosis and implications in acute coronary syndromes. Am J Pathol. 2001;158(3):879-91.

38. Schultz J, Kaminker K. Myeloperoxidase of the leucocyte of normal human blood. I. Content and localization. Arch Biochem Biophys. 1962;96:465-7.

39. Ali M, Pulli B, Courties G, Tricot B, Sebas M, Iwamoto Y, et al. Myeloperoxidase Inhibition Improves Ventricular Function and Remodeling After&#xa0;Experimental Myocardial Infarction. JACC: Basic to Translational Science. 2016;1(7):633-43.

40. Nahrendorf M, Sosnovik D, Chen JW, Panizzi P, Figueiredo J-L, Aikawa E, et al. Activatable magnetic resonance imaging agent reports myeloperoxidase activity in healing infarcts and noninvasively detects the antiinflammatory effects of atorvastatin on ischemia-reperfusion injury. Circulation. 2008;117(9):1153-60.

41. Brown KE, Brunt EM, Heinecke JW. Immunohistochemical detection of myeloperoxidase and its oxidation products in Kupffer cells of human liver. The American journal of pathology. 2001;159(6):2081-8.

42. Daugherty A, Dunn JL, Rateri DL, Heinecke JW. Myeloperoxidase, a catalyst for lipoprotein oxidation, is expressed in human atherosclerotic lesions. J Clin Invest. 1994;94(1):437-44.

43. Gaspar D, Freire JM, Pacheco TR, Barata JT, Castanho MA. Apoptotic human neutrophil peptide-1 anti-tumor activity revealed by cellular biomechanics. Biochimica et Biophysica Acta (BBA)-Molecular Cell Research. 2015;1853(2):308-16.

44. Yomogida S, Nagaoka I, Saito K, Yamashita T. Evaluation of the effects of defensins on neutrophil functions. Inflammation Research. 1996;45(2):62-7.

45. Chertov O, Michiel DF, Xu L, Wang JM, Tani K, Murphy WJ, et al. Identification of Defensin-1, Defensin-2, and CAP37/Azurocidin as T-cell Chemoattractant Proteins Released from Interleukin-8-stimulated Neutrophils (∗). Journal of Biological Chemistry. 1996;271(6):2935-40.

46. Territo MC, Ganz T, Selsted ME, Lehrer R. Monocyte-chemotactic activity of defensins from human neutrophils. J Clin Invest. 1989;84(6):2017-20.

47. Chaly YV, Paleolog EM, Kolesnikova TS, Tikhonov, II, Petratchenko EV, Voitenok NN. Neutrophil alpha-defensin human neutrophil peptide modulates cytokine production in human monocytes and adhesion molecule expression in endothelial cells. Eur Cytokine Netw. 2000;11(2):257-66.

48. Kougias P, Chai H, Lin PH, Yao Q, Lumsden AB, Chen C. Neutrophil antimicrobial peptide α-defensin causes endothelial dysfunction in porcine coronary arteries. Journal of Vascular Surgery. 2006;43(2):357-63.

49. Horn M, Bertling A, Brodde MF, Müller A, Roth J, Van Aken H, et al. Human neutrophil alpha-defensins induce formation of fibrinogen and thrombospondin-1 amyloid-like structures and activate platelets via glycoprotein IIb/IIIa. J Thromb Haemost. 2012;10(4):647-61.

50. Quinn KL, Henriques M, Tabuchi A, Han B, Yang H, Cheng WE, et al. Human neutrophil peptides mediate endothelial-monocyte interaction, foam cell formation, and platelet activation. Arterioscler Thromb Vasc Biol. 2011;31(9):2070-9.

51. Higazi AA, Lavi E, Bdeir K, Ulrich AM, Jamieson DG, Rader DJ, et al. Defensin stimulates the binding of lipoprotein (a) to human vascular endothelial and smooth muscle cells. Blood. 1997;89(12):4290-8.

52. Ganz T. Extracellular release of antimicrobial defensins by human polymorphonuclear leukocytes. Infect Immun. 1987;55(3):568-71.

53. Wilde CG, Griffith JE, Marra MN, Snable JL, Scott RW. Purification and characterization of human neutrophil peptide 4, a novel member of the defensin family. J Biol Chem. 1989;264(19):11200-3.

54. Lehrer RI, Lu W. α‐Defensins in human innate immunity. Immunological reviews. 2012;245(1):84-112.

55. Agerberth B, Charo J, Werr J, Olsson B, Idali F, Lindbom L, et al. The human antimicrobial and chemotactic peptides LL-37 and α-defensins are expressed by specific lymphocyte and monocyte populations. Blood. 2000;96(9):3086-93.

56. Segal AW. How neutrophils kill microbes. Annu Rev Immunol. 2005;23:197-223.

57. Chertov O, Ueda H, Xu LL, Tani K, Murphy WJ, Wang JM, et al. Identification of Human Neutrophil-derived Cathepsin G and Azurocidin/CAP37 as Chemoattractants for Mononuclear Cells and Neutrophils. Journal of Experimental Medicine. 1997;186(5):739-47.

58. Richter R, Bistrian R, Escher S, Forssmann W-G, Vakili J, Henschler R, et al. Quantum Proteolytic Activation of Chemokine CCL15 by Neutrophil Granulocytes Modulates Mononuclear Cell Adhesiveness1. The Journal of Immunology. 2005;175(3):1599-608.

59. Faraday N, Schunke K, Saleem S, Fu J, Wang B, Zhang J, et al. Cathepsin G-dependent modulation of platelet thrombus formation in vivo by blood neutrophils. PLoS One. 2013;8(8):e71447.

60. Korkmaz B, Horwitz MS, Jenne DE, Gauthier F. Neutrophil elastase, proteinase 3, and cathepsin G as therapeutic targets in human diseases. Pharmacol Rev. 2010;62(4):726-59.

61. Yang D, de la Rosa G, Tewary P, Oppenheim JJ. Alarmins link neutrophils and dendritic cells. Trends in immunology. 2009;30(11):531-7.

62. Yan L, Borregaard N, Kjeldsen L, Moses MA. The high molecular weight urinary matrix metalloproteinase (MMP) activity is a complex of gelatinase B/MMP-9 and neutrophil gelatinase-associated lipocalin (NGAL): modulation of MMP-9 activity by NGAL. Journal of Biological Chemistry. 2001;276(40):37258-65.

63. Yan L, Borregaard N, Kjeldsen L, Moses MA. The High Molecular Weight Urinary Matrix Metalloproteinase (MMP) Activity Is a Complex of Gelatinase B/MMP-9 and Neutrophil Gelatinase-associated Lipocalin (NGAL): MODULATION OF MMP-9 ACTIVITY BY NGAL*. Journal of Biological Chemistry. 2001;276(40):37258-65.

64. Wang Y. Small lipid-binding proteins in regulating endothelial and vascular functions: focusing on adipocyte fatty acid binding protein and lipocalin-2. British Journal of Pharmacology. 2012;165(3):603-21.

65. te Boekhorst BC, Bovens SM, Hellings WE, van der Kraak PH, van de Kolk KW, Vink A, et al. Molecular MRI of murine atherosclerotic plaque targeting NGAL: a protein associated with unstable human plaque characteristics. Cardiovascular Research. 2010;89(3):680-8.

66. Folkesson M, Kazil M, Zhu C, Silveira A, Hemdahl A-L, Hamsten A, et al. Presence of NGAL/MMP-9 complexes in human abdominal aortic aneurysms. Thrombosis and haemostasis. 2007;98(08):427-33.

67. Hemdahl AL, Gabrielsen A, Zhu C, Eriksson P, Hedin U, Kastrup J, et al. Expression of neutrophil gelatinase-associated lipocalin in atherosclerosis and myocardial infarction. Arterioscler Thromb Vasc Biol. 2006;26(1):136-42.

68. Passov A, Ilmakunnas M, Pihlajoki M, Hermunen K, Lempinen M, Helanterä I, et al. Neutrophil gelatinase-associated lipocalin does not originate from the kidney during reperfusion in clinical renal transplantation. Intensive Care Med Exp. 2021;9(1):56.

69. Liu F, Yang H, Chen H, Zhang M, Ma Q. High expression of neutrophil gelatinase-associated lipocalin (NGAL) in the kidney proximal tubules of diabetic rats. Adv Med Sci. 2015;60(1):133-8.

70. Yndestad A, Landrø L, Ueland T, Dahl CP, Flo TH, Vinge LE, et al. Increased systemic and myocardial expression of neutrophil gelatinase-associated lipocalin in clinical and experimental heart failure. Eur Heart J. 2009;30(10):1229-36.

71. Marques FZ, Prestes PR, Byars SG, Ritchie SC, Würtz P, Patel SK, et al. Experimental and Human Evidence for Lipocalin-2 (Neutrophil Gelatinase-Associated Lipocalin [NGAL]) in the Development of Cardiac Hypertrophy and heart failure. J Am Heart Assoc. 2017;6(6).

72. Crouch SP, Slater KJ, Fletcher J. Regulation of cytokine release from mononuclear cells by the iron-binding protein lactoferrin. Blood. 1992;80(1):235-40.

73. Bournazou I, Pound JD, Duffin R, Bournazos S, Melville LA, Brown SB, et al. Apoptotic human cells inhibit migration of granulocytes via release of lactoferrin. The Journal of clinical investigation. 2009;119(1):20-32.

74. de la Rosa G, Yang D, Tewary P, Varadhachary A, Oppenheim JJ. Lactoferrin acts as an alarmin to promote the recruitment and activation of APCs and antigen-specific immune responses. J Immunol. 2008;180(10):6868-76.

75. Soehnlein O, Lindbom L. Phagocyte partnership during the onset and resolution of inflammation. Nat Rev Immunol. 2010;10(6):427-39.

76. Baveye S, Elass E, Fernig DG, Blanquart C, Mazurier J, Legrand D. Human lactoferrin interacts with soluble CD14 and inhibits expression of endothelial adhesion molecules, E-selectin and ICAM-1, induced by the CD14-lipopolysaccharide complex. Infect Immun. 2000;68(12):6519-25.

77. Bournazou I, Pound JD, Duffin R, Bournazos S, Melville LA, Brown SB, et al. Apoptotic human cells inhibit migration of granulocytes via release of lactoferrin. J Clin Invest. 2009;119(1):20-32.

78. Bournazou I, Mackenzie KJ, Duffin R, Rossi AG, Gregory CD. Inhibition of eosinophil migration by lactoferrin. Immunol Cell Biol. 2010;88(2):220-3.

79. Scott MG, Davidson DJ, Gold MR, Bowdish D, Hancock RE. The human antimicrobial peptide LL-37 is a multifunctional modulator of innate immune responses. J Immunol. 2002;169(7):3883-91.

80. Herster F, Bittner Z, Archer NK, Dickhöfer S, Eisel D, Eigenbrod T, et al. Neutrophil extracellular trap-associated RNA and LL37 enable self-amplifying inflammation in psoriasis. Nat Commun. 2020;11(1):105.

81. De Y, Chen Q, Schmidt AP, Anderson GM, Wang JM, Wooters J, et al. LL-37, the neutrophil granule- and epithelial cell-derived cathelicidin, utilizes formyl peptide receptor-like 1 (FPRL1) as a receptor to chemoattract human peripheral blood neutrophils, monocytes, and T cells. J Exp Med. 2000;192(7):1069-74.

82. Yang D, Chen Q, Schmidt AP, Anderson GM, Wang JM, Wooters J, et al. LL-37, the Neutrophil Granule–And Epithelial Cell–Derived Cathelicidin, Utilizes Formyl Peptide Receptor–Like 1 (Fprl1) as a Receptor to Chemoattract Human Peripheral Blood Neutrophils, Monocytes, and T Cells. Journal of Experimental Medicine. 2000;192(7):1069-74.

83. van der Does AM, Beekhuizen H, Ravensbergen B, Vos T, Ottenhoff THM, van Dissel JT, et al. LL-37 Directs Macrophage Differentiation toward Macrophages with a Proinflammatory Signature. The Journal of Immunology. 2010;185(3):1442-9.

84. Koczulla R, von Degenfeld G, Kupatt C, Krötz F, Zahler S, Gloe T, et al. An angiogenic role for the human peptide antibiotic LL-37/hCAP-18. J Clin Invest. 2003;111(11):1665-72.

85. Carretero M, Escámez MJ, García M, Duarte B, Holguín A, Retamosa L, et al. In vitro and in vivo wound healing-promoting activities of human cathelicidin LL-37. J Invest Dermatol. 2008;128(1):223-36.

86. Nagaoka I, Tamura H, Hirata M. An antimicrobial cathelicidin peptide, human CAP18/LL-37, suppresses neutrophil apoptosis via the activation of formyl-peptide receptor-like 1 and P2X7. J Immunol. 2006;176(5):3044-52.

87. Sørensen OE, Follin P, Johnsen AH, Calafat J, Tjabringa GS, Hiemstra PS, et al. Human cathelicidin, hCAP-18, is processed to the antimicrobial peptide LL-37 by extracellular cleavage with proteinase 3. Blood. 2001;97(12):3951-9.

88. De Y, Chen Q, Schmidt AP, Anderson GM, Wang JM, Wooters J, et al. LL-37, the neutrophil granule- and epithelial cell-derived cathelicidin, utilizes formyl peptide receptor-like 1 (FPRL1) as a receptor to chemoattract human peripheral blood neutrophils, monocytes, and T cells. The Journal of experimental medicine. 2000;192(7):1069-74.

89. Zanetti M. Cathelicidins, multifunctional peptides of the innate immunity. J Leukoc Biol. 2004;75(1):39-48.

90. Edfeldt K, Agerberth B, Rottenberg ME, Gudmundsson GH, Wang XB, Mandal K, et al. Involvement of the antimicrobial peptide LL-37 in human atherosclerosis. Arterioscler Thromb Vasc Biol. 2006;26(7):1551-7.

91. Endo Y, Matsushita M, Fujita T. The role of ficolins in the lectin pathway of innate immunity. The International Journal of Biochemistry & Cell Biology. 2011;43(5):705-12.

92. Pilely K, Rosbjerg A, Genster N, Gal P, Pál G, Halvorsen B, et al. Cholesterol crystals activate the lectin complement pathway via ficolin-2 and mannose-binding lectin: implications for the progression of atherosclerosis. The Journal of Immunology. 2016;196(12):5064-74.

93. Zhang J, Yang L, Ang Z, Yoong SL, Tran TT, Anand GS, et al. Secreted M-ficolin anchors onto monocyte transmembrane G protein-coupled receptor 43 and cross talks with plasma C-reactive protein to mediate immune signaling and regulate host defense. J Immunol. 2010;185(11):6899-910.

94. Fumagalli S, De Simoni M-G. Lectin complement pathway and its bloody interactions in brain ischemia. Stroke. 2016;47(12):3067-73.

95. Rørvig S, Honore C, Larsson LI, Ohlsson S, Pedersen CC, Jacobsen LC, et al. Ficolin-1 is present in a highly mobilizable subset of human neutrophil granules and associates with the cell surface after stimulation with fMLP. J Leukoc Biol. 2009;86(6):1439-49.

96. Honoré C, Rørvig S, Munthe-Fog L, Hummelshøj T, Madsen HO, Borregaard N, et al. The innate pattern recognition molecule Ficolin-1 is secreted by monocytes/macrophages and is circulating in human plasma. Mol Immunol. 2008;45(10):2782-9.

97. Carbone F, Valente A, Perego C, Bertolotto M, Pane B, Spinella G, et al. Ficolin-2 serum levels predict the occurrence of acute coronary syndrome in patients with severe carotid artery stenosis. Pharmacological Research. 2021;166:105462.

98. van den Borne SW, Cleutjens JP, Hanemaaijer R, Creemers EE, Smits JF, Daemen MJ, et al. Increased matrix metalloproteinase-8 and -9 activity in patients with infarct rupture after myocardial infarction. Cardiovasc Pathol. 2009;18(1):37-43.

99. Laxton RC, Hu Y, Duchene J, Zhang F, Zhang Z, Leung K-Y, et al. A role of matrix metalloproteinase-8 in atherosclerosis. Circulation research. 2009;105(9):921-9.

100. Tester AM, Cox JH, Connor AR, Starr AE, Dean RA, Puente XS, et al. LPS Responsiveness and Neutrophil Chemotaxis In Vivo Require PMN MMP-8 Activity. PLoS One. 2007;2(3):e312.

101. Siwik DA, Pagano PJ, Colucci WS. Oxidative stress regulates collagen synthesis and matrix metalloproteinase activity in cardiac fibroblasts. Am J Physiol Cell Physiol. 2001;280(1):C53-60.

102. Sorsa T, Tjäderhane L, Konttinen YT, Lauhio A, Salo T, Lee HM, et al. Matrix metalloproteinases: contribution to pathogenesis, diagnosis and treatment of periodontal inflammation. Ann Med. 2006;38(5):306-21.

103. Lindsey M, Wedin K, Brown MD, Keller C, Evans AJ, Smolen J, et al. Matrix-dependent mechanism of neutrophil-mediated release and activation of matrix metalloproteinase 9 in myocardial ischemia/reperfusion. Circulation. 2001;103(17):2181-7.

104. Luttun A, Lutgens E, Manderveld A, Maris K, Collen D, Carmeliet P, et al. Loss of matrix metalloproteinase-9 or matrix metalloproteinase-12 protects apolipoprotein E–deficient mice against atherosclerotic media destruction but differentially affects plaque growth. Circulation. 2004;109(11):1408-14.

105. Lindsey ML, Escobar GP, Dobrucki LW, Goshorn DK, Bouges S, Mingoia JT, et al. Matrix metalloproteinase-9 gene deletion facilitates angiogenesis after myocardial infarction. Am J Physiol Heart Circ Physiol. 2006;290(1):H232-9.

106. De Nooijer R, Verkleij C, Von der Thusen J, Jukema J, Van der Wall E, van Berkel TJ, et al. Lesional overexpression of matrix metalloproteinase-9 promotes intraplaque hemorrhage in advanced lesions but not at earlier stages of atherogenesis. Arteriosclerosis, thrombosis, and vascular biology. 2006;26(2):340-6.

107. Brown DL, Hibbs MS, Kearney M, Loushin C, Isner JM. Identification of 92-kD Gelatinase in Human Coronary Atherosclerotic Lesions. Circulation. 1995;91(8):2125-31.

108. Galis ZS, Sukhova GK, Lark MW, Libby P. Increased expression of matrix metalloproteinases and matrix degrading activity in vulnerable regions of human atherosclerotic plaques. The Journal of Clinical Investigation. 1994;94(6):2493-503.

109. Lindsey ML, Zamilpa R. Temporal and spatial expression of matrix metalloproteinases and tissue inhibitors of metalloproteinases following myocardial infarction. Cardiovasc Ther. 2012;30(1):31-41.

110. Galis ZS, Khatri JJ. Matrix metalloproteinases in vascular remodeling and atherogenesis: the good, the bad, and the ugly. Circulation research. 2002;90(3):251-62.

111. Chen B, Miller AL, Rebelatto M, Brewah Y, Rowe DC, Clarke L, et al. S100A9 Induced Inflammatory Responses Are Mediated by Distinct Damage Associated Molecular Patterns (DAMP) Receptors In Vitro and In Vivo. PLoS One. 2015;10(2):e0115828.

112. Croce K, Gao H, Wang Y, Mooroka T, Sakuma M, Shi C, et al. Myeloid-related protein-8/14 is critical for the biological response to vascular injury. Circulation. 2009;120(5):427-36.

113. Ryckman C, Vandal K, Rouleau P, Talbot Mv, Tessier PA. Proinflammatory Activities of S100: Proteins S100A8, S100A9, and S100A8/A9 Induce Neutrophil Chemotaxis and Adhesion 1. The Journal of Immunology. 2003;170(6):3233-42.

114. Kerkhoff C, Eue I, Sorg C. The regulatory role of MRP8 (S100A8) and MRP14 (S100A9) in the transendothelial migration of human leukocytes. Pathobiology. 1999;67(5-6):230-2.

115. Lackmann M, Rajasekariah P, Iismaa S, Jones G, Cornish C, Hu S, et al. Identification of a chemotactic domain of the pro-inflammatory S100 protein CP-10. The Journal of Immunology. 1993;150(7):2981-91.

116. Tardif MR, Chapeton-Montes JA, Posvandzic A, Pagé N, Gilbert C, Tessier PA. Secretion of S100A8, S100A9, and S100A12 by neutrophils involves reactive oxygen species and potassium efflux. Journal of immunology research. 2015;2015.

117. Schenten V, Plançon S, Jung N, Hann J, Bueb JL, Bréchard S, et al. Secretion of the Phosphorylated Form of S100A9 from Neutrophils Is Essential for the Proinflammatory Functions of Extracellular S100A8/A9. Front Immunol. 2018;9:447.

118. Cesaro A, Anceriz N, Plante A, Pagé N, Tardif MR, Tessier PA. An Inflammation Loop Orchestrated by S100A9 and Calprotectin Is Critical for Development of Arthritis. PLoS One. 2012;7(9):e45478.

119. Li Y, Chen B, Yang X, Zhang C, Jiao Y, Li P, et al. S100a8/a9 signaling causes mitochondrial dysfunction and cardiomyocyte death in response to ischemic/reperfusion injury. Circulation. 2019;140(9):751-64.

120. Boyd JH, Kan B, Roberts H, Wang Y, Walley KR. S100A8 and S100A9 mediate endotoxin-induced cardiomyocyte dysfunction via the receptor for advanced glycation end products. Circulation research. 2008;102(10):1239-46.

121. Sreejit G, Abdel-Latif A, Athmanathan B, Annabathula R, Dhyani A, Noothi SK, et al. Neutrophil-Derived S100A8/A9 Amplify Granulopoiesis After Myocardial Infarction. Circulation. 2020;141(13):1080-94.

122. Altwegg LA, Neidhart M, Hersberger M, Müller S, Eberli FR, Corti R, et al. Myeloid-related protein 8/14 complex is released by monocytes and granulocytes at the site of coronary occlusion: a novel, early, and sensitive marker of acute coronary syndromes. European Heart Journal. 2007;28(8):941-8.

123. Mahnke K, Bhardwaj R, Sorg C. Heterodimers of the calcium-binding proteins MRP8 and MRP14 are expressed on the surface of human monocytes upon adherence to fibronectin and collagen. Relation to TNF-alpha, IL-6, and superoxide production. J Leukoc Biol. 1995;57(1):63-71.

124. Rammes A, Roth J, Goebeler M, Klempt M, Hartmann M, Sorg C. Myeloid-related protein (MRP) 8 and MRP14, calcium-binding proteins of the S100 family, are secreted by activated monocytes via a novel, tubulin-dependent pathway. J Biol Chem. 1997;272(14):9496-502.

125. Healy AM, Pickard MD, Pradhan AD, Wang Y, Chen Z, Croce K, et al. Platelet expression profiling and clinical validation of myeloid-related protein-14 as a novel determinant of cardiovascular events. Circulation. 2006;113(19):2278-84.

126. Hogg N, Allen C, Edgeworth J. Monoclonal antibody 5.5 reacts with p8,14, a myeloid molecule associated with some vascular endothelium. European Journal of Immunology. 1989;19(6):1053-61.

127. McCormick MM, Rahimi F, Bobryshev YV, Gaus K, Zreiqat H, Cai H, et al. S100A8 and S100A9 in human arterial wall. Implications for atherogenesis. J Biol Chem. 2005;280(50):41521-9.

128. Inaba H, Hokamura K, Nakano K, Nomura R, Katayama K, Nakajima A, et al. Upregulation of S100 calcium-binding protein A9 is required for induction of smooth muscle cell proliferation by a periodontal pathogen. FEBS Lett. 2009;583(1):128-34.

129. Mangold A, Hofbauer TM, Ondracek AS, Artner T, Scherz T, Speidl WS, et al. Neutrophil extracellular traps and monocyte subsets at the culprit lesion site of myocardial infarction patients. Scientific Reports. 2019;9(1):16304.

130. Döring Y, Soehnlein O, Weber C. Neutrophil Extracellular Traps in Atherosclerosis and Atherothrombosis. Circ Res. 2017;120(4):736-43.

131. Lande R, Ganguly D, Facchinetti V, Frasca L, Conrad C, Gregorio J, et al. Neutrophils activate plasmacytoid dendritic cells by releasing self-DNA-peptide complexes in systemic lupus erythematosus. Sci Transl Med. 2011;3(73):73ra19.

132. Garcia-Romo GS, Caielli S, Vega B, Connolly J, Allantaz F, Xu Z, et al. Netting neutrophils are major inducers of type I IFN production in pediatric systemic lupus erythematosus. Sci Transl Med. 2011;3(73):73ra20.

133. de Boer OJ, Li X, Teeling P, Mackaay C, Ploegmakers HJ, van der Loos CM, et al. Neutrophils, neutrophil extracellular traps and interleukin-17 associate with the organisation of thrombi in acute myocardial infarction. Thromb Haemost. 2013;109(2):290-7.

134. Sanchis J, García-Blas S, Ortega-Paz L, Dantas AP, Rodríguez E, Abellán L, et al. Cell-free DNA and microvascular damage in ST-segment elevation myocardial infarction treated with primary percutaneous coronary intervention. Revista Española de Cardiología (English Edition). 2019;72(4):317-23.

135. Branzk N, Lubojemska A, Hardison SE, Wang Q, Gutierrez MG, Brown GD, et al. Neutrophils sense microbe size and selectively release neutrophil extracellular traps in response to large pathogens. Nat Immunol. 2014;15(11):1017-25.

136. von Köckritz-Blickwede M, Goldmann O, Thulin P, Heinemann K, Norrby-Teglund A, Rohde M, et al. Phagocytosis-independent antimicrobial activity of mast cells by means of extracellular trap formation. Blood. 2008;111(6):3070-80.

137. Yousefi S, Mihalache C, Kozlowski E, Schmid I, Simon H-U. Viable neutrophils release mitochondrial DNA to form neutrophil extracellular traps. Cell Death & Differentiation. 2009;16(11):1438-44.

138. Gasser O, Hess C, Miot S, Deon C, Sanchez JC, Schifferli JA. Characterisation and properties of ectosomes released by human polymorphonuclear neutrophils. Exp Cell Res. 2003;285(2):243-57.

139. Loyer X, Vion AC, Tedgui A, Boulanger CM. Microvesicles as cell-cell messengers in cardiovascular diseases. Circ Res. 2014;114(2):345-53.

140. Warnatsch A, Ioannou M, Wang Q, Papayannopoulos V. Inflammation. Neutrophil extracellular traps license macrophages for cytokine production in atherosclerosis. Science. 2015;349(6245):316-20.

141. Eken C, Gasser O, Zenhaeusern G, Oehri I, Hess C, Schifferli JrA. Polymorphonuclear Neutrophil-Derived Ectosomes Interfere with the Maturation of Monocyte-Derived Dendritic Cells1. The Journal of Immunology. 2008;180(2):817-24.

142. Mallat Z, Benamer H, Hugel B, Benessiano J, Steg PG, Freyssinet JM, et al. Elevated levels of shed membrane microparticles with procoagulant potential in the peripheral circulating blood of patients with acute coronary syndromes. Circulation. 2000;101(8):841-3.

143. Wang JG, Williams JC, Davis BK, Jacobson K, Doerschuk CM, Ting JP, et al. Monocytic microparticles activate endothelial cells in an IL-1β-dependent manner. Blood. 2011;118(8):2366-74.

144. Pitanga TN, de Aragão França L, Rocha VC, Meirelles T, Borges VM, Gonçalves MS, et al. Neutrophil-derived microparticles induce myeloperoxidase-mediated damage of vascular endothelial cells. BMC Cell Biol. 2014;15:21.

145. Min PK, Kim JY, Chung KH, Lee BK, Cho M, Lee DL, et al. Local increase in microparticles from the aspirate of culprit coronary arteries in patients with ST-segment elevation myocardial infarction. Atherosclerosis. 2013;227(2):323-8.

146. Baldus SH, C.; Meinertz, T.; Zeiher, A. M.; Eiserich, J. P.; Munzel, T.; Simoons, M. L.; Hamm, C. W. Myeloperoxidase serum levels predict risk in patients with acute coronary syndromes. Circulation. 2003;108(12):1440-5.

147. Brügger-Andersen T, Aarsetøy H, Grundt H, Staines H, Nilsen DWT. The long-term prognostic value of multiple biomarkers following a myocardial infarction. Thrombosis Research. 2008;123(1):60-6.

148. Cavusoglu E, Ruwende C, Eng C, Chopra V, Yanamadala S, Clark LT, et al. Usefulness of Baseline Plasma Myeloperoxidase Levels as an Independent Predictor of Myocardial Infarction at Two Years in Patients Presenting With Acute Coronary Syndrome. The American Journal of Cardiology. 2007;99(10):1364-8.

149. McCann CJ, Glover BM, Menown IBA, Moore MJ, McEneny J, Owens CG, et al. Prognostic Value of a Multimarker Approach for Patients Presenting to Hospital With Acute Chest Pain. The American Journal of Cardiology. 2009;103(1):22-8.

150. Mocatta TJ, Pilbrow AP, Cameron VA, Senthilmohan R, Frampton CM, Richards AM, et al. Plasma concentrations of myeloperoxidase predict mortality after myocardial infarction. J Am Coll Cardiol. 2007;49(20):1993-2000.

151. Nicholls SJ, Hazen SL. Myeloperoxidase and Cardiovascular Disease. Arteriosclerosis, Thrombosis, and Vascular Biology. 2005;25(6):1102-11.

152. Scirica BM, Sabatine MS, Jarolim P, Murphy SA, de Lemos JL, Braunwald E, et al. Assessment of multiple cardiac biomarkers in non-ST-segment elevation acute coronary syndromes: observations from the MERLIN-TIMI 36 Trial. European Heart Journal. 2010;32(6):697-705.

153. Brennan MP, M. S.; Van Lente, F.; Nambi, V.; Shishehbor, M. H.; Aviles, R. J.; Goormastic, M.; Pepoy, M. L.; McErlean, E. S.; Topol, E. J.; Nissen, S. E.; Hazen, S. L. Prognostic value of myeloperoxidase in patients with chest pain. New England Journal of Medicine. 2003;349(17):1595-604.

154. Apple FS, Smith SW, Pearce LA, Schulz KM, Ler R, Murakami MM. Myeloperoxidase Improves Risk Stratification in Patients with Ischemia and Normal Cardiac Troponin I Concentrations. Clinical Chemistry. 2011;57(4):603-8.

155. Kaya MG, Yalcin R, Okyay K, Poyraz F, Bayraktar N, Pasaoglu H, et al. Potential role of plasma myeloperoxidase level in predicting long-term outcome of acute myocardial infarction. Tex Heart Inst J. 2012;39(4):500-6.

156. Roman RM, Camargo PV, Borges FK, Rossini AP, Polanczyk CA. Prognostic value of myeloperoxidase in coronary artery disease: comparison of unstable and stable angina patients. Coron Artery Dis. 2010;21(3):129-36.

157. Scharnagl H, Kleber ME, Genser B, Kickmaier S, Renner W, Weihrauch G, et al. Association of myeloperoxidase with total and cardiovascular mortality in individuals undergoing coronary angiography—The LURIC study. International Journal of Cardiology. 2014;174(1):96-105.

158. Giurgea GA, Zlabinger K, Gugerell A, Lukovic D, Syeda B, Mandic L, et al. Multimarker Approach to Identify Patients with Coronary Artery Disease at High Risk for Subsequent Cardiac Adverse Events: The Multi-Biomarker Study. Biomolecules. 2020;10(6).

159. Helanova KL, S.; Kubena, P.; Ganovska, E.; Pavlusova, M.; Kubkova, L.; Jarkovsky, J.; Goldbergova, M. P.; Lipkova, J.; Gottwaldova, J.; Kala, P.; Toman, O.; Dastych, M.; Spinar, J.; Parenica, J. Prognostic impact of neutrophil gelatinase-associated lipocalin and B-type natriuretic in patients with ST-elevation myocardial infarction treated by primary PCI: A prospective observational cohort study. BMJ Open. 2015;5(10) (no pagination).

160. Langseth MSH, R.; Ritschel, V.; Hansen, C. H.; Andersen, G. O.; Eritsland, J.; Halvorsen, S.; Fagerland, M. W.; Solheim, S.; Arnesen, H.; Seljeflot, I.; Opstad, T. B. Double-Stranded DNA and NETs Components in Relation to Clinical Outcome After ST-Elevation Myocardial Infarction. Scientific Reports. 2020;10(1):5007.

161. Helseth R, Shetelig C, Andersen GØ, Langseth MS, Limalanathan S, Opstad TB, et al. Neutrophil Extracellular Trap Components Associate with Infarct Size, Ventricular Function, and Clinical Outcome in STEMI. Mediators of Inflammation. 2019;2019:7816491.

162. Morrow DA, Sabatine MS, Brennan M-L, de Lemos JA, Murphy SA, Ruff CT, et al. Concurrent evaluation of novel cardiac biomarkers in acute coronary syndrome: myeloperoxidase and soluble CD40 ligand and the risk of recurrent ischaemic events in TACTICS-TIMI 18. European Heart Journal. 2008;29(9):1096-102.

163. Rainer TH, Lam NY, Man CY, Chiu RW, Woo KS, Lo YM. Plasma beta-globin DNA as a prognostic marker in chest pain patients. Clin Chim Acta. 2006;368(1-2):110-3.

164. Nymo SHH, M.; Ueland, T.; Yndestad, A.; Lorentzen, E.; Truvé, K.; Karlsson, T.; Ravn-Fischer, A.; Aukrust, P.; Caidahl, K. Serum neutrophil gelatinase-associated lipocalin (NGAL) concentration is independently associated with mortality in patients with acute coronary syndrome. International Journal of Cardiology. 2018;262:79-84.

165. Katagiri M, Takahashi M, Doi K, Myojo M, Kiyosue A, Ando J, et al. Serum neutrophil gelatinase-associated lipocalin concentration reflects severity of coronary artery disease in patients without heart failure and chronic kidney disease. Heart Vessels. 2016;31(10):1595-602.

166. Liu J, Yang D, Wang X, Zhu Z, Wang T, Ma A, et al. Neutrophil extracellular traps and dsDNA predict outcomes among patients with ST-elevation myocardial infarction. Scientific Reports. 2019;9(1):11599.

167. Avci AO, B.; Demir, K.; Akyurek, F.; Altunkeser, B. B. The Prognostic Utility of Plasma NGAL Levels in ST Segment Elevation in Myocardial Infarction Patients. Advances in Preventive Medicine. 2020;2020:4637043.

168. Obeid SY, N.; Davies, A.; Loretz, R.; Saleh, L.; Niederseer, D.; Noor, H. A.; Amin, H.; Mach, F.; Gencer, B.; Raber, L.; Windecker, S.; Templin, C.; Nanchen, D.; Rodondi, N.; Muller, O.; Matter, C. M.; von Eckardstein, A.; Luscher, T. F. Prognostic role of plasma galectin-3 levels in acute coronary syndrome. European Heart Journal: Acute Cardiovascular Care. 2020;9(8):869-78.

169. Peng WZ, C.; Wang, Z.; Yang, W.; Luo, H.; Li, X.; Fu, D.; Yu, C.; Zhou, Y. Prognostic value of neutrophil gelatinase-Associated lipocalin and glycosylated hemoglobin for non-ST-segment elevation myocardial infarction patients with single concomitant chronic total occlusion following primary percutaneous coronary intervention: A prospective observational study. Medicine. 2019;98(39).

170. Nguyen LSS, V.; Kerneis, M.; Hauguel-Moreau, M.; Barthélémy, O.; Collet, J. P.; Montalescot, G.; Silvain, J. Evaluation of neutrophil gelatinase-associated lipocalin and cystatin C as biomarkers of acute kidney injury after ST-segment elevation myocardial infarction treated by percutaneous coronary intervention. Arch Cardiovasc Dis. 2019;112(3):180-6.

171. Wang CJK, Y.; Ding, Y. Y.; Sun, J. Z.; Chen, T. Serum Calprotectin Levels and Outcome Following Percutaneous Coronary Intervention in Patients with Diabetes and Acute Coronary Syndrome. Medical Science Monitor. 2019;25:9517-23.

172. Wang XY, D.; Liu, J.; Fan, X.; Ma, A.; Liu, P. Prognostic value of culprit artery double-stranded DNA in ST-segment elevated myocardial infarction. Scientific Reports. 2018;8(1):9294.

173. Barbarash OLB, I. S.; Kashtalap, V. V.; Zykov, M. V.; Hryachkova, O. N.; Kalaeva, V. V.; Shafranskaya, K. S.; Karetnikova, V. N.; Kutikhin, A. G. Serum neutrophil gelatinase-associated lipocalin has an advantage over serum cystatin C and glomerular filtration rate in prediction of adverse cardiovascular outcome in patients with ST-segment elevation myocardial infarction. BMC Cardiovascular Disorders. 2017;17(1):81.

174. Akcay ABO, M. F.; Sen, N.; Cay, S.; Ozturk, O. H.; Yalcn, F.; Bilen, P.; Kanat, S.; Karakas, M. F.; Isleyen, A.; Demir, A. D.; Sogut, S.; Covic, A.; Kanbay, M. Prognostic significance of neutrophil gelatinase-associated lipocalin in ST-segment elevation myocardial infarction. Journal of Investigative Medicine. 2012;60(2):508-13.

175. Alfakry HS, J.; Paju, S.; Nieminen, M. S.; Valtonen, V.; Tervahartiala, T.; Pussinen, P. J.; Sorsa, T. The association of serum neutrophil markers and acute coronary syndrome. Scand J Immunol. 2012;76(2):181-7.

176. Lindberg SP, S. H.; Mogelvang, R.; Jensen, J. S.; Flyvbjerg, A.; Galatius, S.; Magnusson, N. E. Prognostic utility of neutrophil gelatinase-associated lipocalin in predicting mortality and cardiovascular events in patients with ST-segment elevation myocardial infarction treated with primary percutaneous coronary intervention. Journal of the American College of Cardiology. 2012;60(4):339-45.

177. Ng LLK, S. Q.; Narayan, H.; Quinn, P.; Squire, I. B.; Davies, J. E. Proteinase 3 and prognosis of patients with acute myocardial infarction. Clinical Science. 2011;120(6):231-8.

178. Jensen LJNP, S.; Bjerre, M.; Mogelvang, R.; Jensen, J. S.; Flyvbjerg, A. Plasma Calprotectin Predicts Mortality in Patients with ST Segment Elevation Myocardial Infarction Treated with Primary Percutaneous Coronary Intervention. Journal of Interventional Cardiology. 2010;23(2):123-9.

179. Hally KEP, O. M.; Brunton-O'sullivan M, M.; Harding, S. A.; Larsen, P. D. Linking Neutrophil Extracellular Traps and Platelet Activation: A Composite Biomarker Score for Predicting Outcomes after Acute Myocardial Infarction. Thrombosis and Haemostasis. 2021.
